# The physicochemical fingerprint of *Necator americanus*

**DOI:** 10.1371/journal.pntd.0005971

**Published:** 2017-12-07

**Authors:** Veeren M. Chauhan, David J. Scurr, Thomas Christie, Gary Telford, Jonathan W. Aylott, David I. Pritchard

**Affiliations:** School of Pharmacy, Boots Science Building, University of Nottingham, Nottingham, United Kingdom; Universidade Federal de Minas Gerais, BRAZIL

## Abstract

*Necator americanus*, a haematophagous hookworm parasite, infects ~10% of the world’s population and is considered to be a significant public health risk. Its lifecycle has distinct stages, permitting its successful transit from the skin *via* the lungs (L_3_) to the intestinal tract (L_4_ maturing to adult). It has been hypothesised that the L_3_ larval sheath, which is shed during percutaneous infection (exsheathment), diverts the immune system to allow successful infection and reinfection in endemic areas. However, the physicochemical properties of the L_3_ larval cuticle and sheath, which are in direct contact with the skin and its immune defences, are unknown. In the present study, we controlled exsheathment, to characterise the sheath and underlying cuticle surfaces *in situ*, using atomic force microscopy (AFM) and time-of-flight secondary ion mass spectrometry (ToF-SIMS). AFM revealed previously unseen surface area enhancing nano-annuli exclusive to the sheath surface and confirmed greater adhesion forces exist between cationic surfaces and the sheath, when compared to the emergent L_3_ cuticle. Furthermore, ToF-SIMS elucidated different chemistries between the surfaces of the cuticle and sheath which could be of biological significance. For example, the phosphatidylglycerol rich cuticle surface may support the onward migration of a lubricated infective stage, while the anionic and potentially immunologically active heparan sulphate rich deposited sheath could result in the diversion of immune defences to an inanimate antigenic nidus. We propose that our initial studies into the surface analysis of this hookworm provides a timely insight into the physicochemical properties of a globally important human pathogen at its infective stage and anticipate that the development and application of this analytical methodology will support translation of these findings into a biological context.

## Author summary

*Necator americanus* is an intestinal hookworm parasite of humans that is commonly found in tropical and sub-tropical climates. *N*. *americanus* infections can be treated effectively with anthelmintic drug therapy; however, in endemic areas re-infection quickly returns. Chronic hookworm infection can lead to intestinal blood loss, iron deficiency anaemia, malnutrition and physical and intellectual impairment. *N*. *americanus* surfaces may possess key physicochemical properties that permit successful host infection. Therefore, we harnessed controlled exsheathment of infective axenic L_3_ larva to investigate the physicochemical properties of the emergent cuticle and deposited sheath, using atomic force microscopy (AFM) and time-of-flight secondary ion mass spectrometry (ToF-SIMS). Our results provide an early insight into the differential physicochemical properties of these bio-surfaces, allowing the development of a hypothesis as to how these chemistries may be involved in infection and immune evasion. This new analytical platform will allow us to test this hypothesis and translate our findings into an immuno-biological context.

## Introduction

*Necator americanus*, the “American Murderer” [1], is a hookworm parasite of humans [2]. Infection occurs in areas of poor hygiene and sanitation when individuals come into contact with soil containing the faecal matter of infected hosts [3].

Fertilised eggs hatch in the faecal mass and the emerging larvae develop and moult from a larval L_1_ stage to an infective L_3_ stage (~ 10 days). The L_3_ stage is protected from desiccation in tropical climates by a sheath, which is formed from the shed cuticle of the L_2_ developmental stage [4]. This permits the infective L_3_ stage to survive long enough to support an encounter with the next human host in the transmission cycle. When the ensheathed L_3_ encounter human skin surfaces stimulatory cues heighten nictitating behaviour, which are thought to promote exsheathment [5] and transdermal invasion [6]. Exsheathed L_3_ resume development in the skin before they transit to the lungs, where they migrate from the airways and are swallowed into the gastrointestinal tract [7]. During this migratory period the larvae mature to the L_4_ stage. On arrival to the small intestine, *N*. *americanus* attach to the intestinal mucosa and feed on host mucosa and blood [8], which supports their development into separately sexed adults. Mated females produce progeny in the form of fertilised eggs, which are transferred with the faeces on to the soil. This completes and facilitates the continuation of the hookworm life-cycle [9].

The hookworm life-cycle is very successful with an estimated 576–700 million people infected worldwide [10]. Individuals with a light hookworm burden are usually asymptomatic, while hosts with a heavy hookworm burden present with symptoms of ‘hookworm disease,’ which include anaemia, fever, diarrhoea, nausea, vomiting, rashes, abdominal pain and intestinal cramps [11, 12]. Untreated chronic infections can cause long term discomfort and disability, with patients exhibiting lassitude and peripheral oedema [13, 14]. These symptoms are caused by the haematophagous capacity of the hookworm [15] supported by the secretion of defined anti-haemostatic molecules [16].

Hookworm infections can be adequately controlled in the short term through use of anthelmintic drugs such as mebendazole, albendazole, and pyrantel pamoate [17]. However, for individuals inhabiting endemic areas, successful drug treatment is ephemeral, as infections quickly return post-treatment and can persist throughout a lifetime [18]. Drug resistance can be problematic [19] and reinfection may occur due to an absence of effective immunity [20]. Nevertheless, the allergic phenotype, characterised by elevated IgE, IL5 and eosinophilia, appear to have a controlling influence over infecting hookworms, which could effect on their ability to feed and contribute to the reduction in adult hookworm size and fecundity [21].

At this point, it is important to note that the absence of effective immunity is not due to the failure of the immune system to recognise the parasite, as immunological responses are detectable at all parasitic stages [22, 23]. Therefore, it has been postulated that *N*. *americanus* utilises a number of complementary immune evasion strategies to invade, survive and maintain its successful colonisation of human hosts [24]. Research into the host evasion strategies are diverse [25–27], but have concentrated on the later life cycle stages of helminth infections, in particular the role of adult hookworm secretions in manipulating the immune response [28]. More recently, *N*. *americanus* have been shown to effectively control autoimmune enteropathy at the site of infection [29]. Despite these advances in understanding of hookworm infection, little is known of the percutaneous infection process in humans, and its contribution to immune evasion and reinfection.

In the present work we develop a hypothesis that focuses on the primary stages of *N*. *americanus* infection and reinfection. In animal models, L_3_ larvae remain in the skin for approximately 48 hours [30]. During this period, they receive environmental cues to resume feeding, prior to their migration to the lungs. Should this occur in humans, this period of residence in the skin would provide the immune system with ample opportunity to arrest larval development, at what is considered to be a highly immunologically active anatomical site. However, as reinfection regularly occurs in endemic areas, this strongly suggests that the immune system fails to effectively target the infective L_3_ larva. We propose that during infection the deposited larval sheath functions to divert the immune system away from the emerging and migrating L_3_ larva, a phenomenon achieved as a result of differences in both physical and chemical surface properties of the emergent cuticle and the sheath.

This hypothesis is dependent on evidence that the sheath is deposited in the skin during infection. Although the exact sequence of events during infection in humans is unknown, previously published *ex vivo* work shows; I) surface fluoresceinated ensheathed L_3_ present in the dermal layers and subcutaneous adipose tissue following infection [6], only 30% of the larval sheaths were recoverable from the skin surface in these experiments, suggesting that 70% had been carried into the skin, II) an enduring pruritic erythematous papular rash at the site of infection suggesting the presence of an antigenic nidus, III) the age-related presence of circulating antibodies in 203 infected humans to cetyltrimethylammonium bromide stripped sheath proteins and to exsheathing fluid [6], and IV) the age-related appearance of antibodies to collagen, a major component of L_3_ larval sheath [31, 32]. These age-related profiles could be indicative of an acquired immune response in humans to repeated sheath deposition, although antigenic cross reactivity cannot be discounted with later moulting life cycle stages. These observations cannot be totally supportive of the hypothesis given our current knowledge of infection in humans, yet they are highly suggestive of antigenic sheath deposition at an immunologically active site. Conclusive evidence will only come from human volunteer studies, where skin biopsies will be required to fully investigate this phase of the infection process.

We postulate that the physicochemical properties of the cuticle and sheath will be fundamentally different, to the extent that any observed differences could contribute to immune evasion as well as the biological process of larval migration. These chemical differences were alluded to in early electrophoretic experiments [33], where iodination of the sheath and cuticle produced significantly different profiles on subsequent SDS-PAGE analysis. In these experiments the sheath was diffusely labelled, which indicates of a high degree of glycosylation. In contrast, the cuticle demonstrates a defined labelling profile, indicating the presence of discrete proteins.

To understand the physicochemical properties of nematode surfaces further, studies have also used surface labels and complementary optical techniques to investigate topography [34] and produced nematode homogenates, which have been examined with liquid chromatography mass spectrometry (LC-MS) to determine chemical characteristics [35]. However, surface labels can generate optical artefacts when interpreting the data and homogenates can often produce uncertainty as to whether the chemical entities are present on the surface, as the body would observe their presence, or distributed throughout the homogenate.

To investigate the physical and chemical properties of the emerging L_3_ cuticle and sheath we initially studied the behaviour of ensheathed larvae to enhance our understanding of the exsheathment process. The surface topography of partially exsheathed *N*. *americanus* was primarily investigated using optical microscopy and environmental scanning electron microscopy (ESEM). This was followed by an in depth analysis of sheath and cuticle height using atomic force microscopy (AFM)[36] and quantitative nanomechanical mapping (QNM), which was employed to determine surface adhesive forces. The surface chemistries of the partially exsheathed L_3_ larvae were directly ascertained using time-of-flight secondary ion mass spectrometry (ToF-SIMS)[37]. Chemical similarities and dissimilarities between the L_3_ cuticle and sheath were identified using a combination of statistical analyses, which included principal component analysis (PCA) and multivariate curve resolution (MCR) analysis.

## Results/Discussion

### Controlled exsheathment

In order to examine the surface properties of the L_3_ cuticle and sheath the larvae are required to be exsheathed in a controlled manner rendering them amenable to analysis. Therefore, the exsheathment process was investigated through deposition of axenic larvae, produced according to cGMP standards and deemed to be free of bacterial contamination, on either clean glass slides or glass slides freshly coated with poly-L-lysine, whilst being subjected to relatively high (37°C) and low (20°C) temperatures. Poly-L-lysine is a polycationic molecule that can be used to coat solid surfaces, such as glass, providing sites of adhesion for biological systems through interaction with anionic crypts present on biological surfaces. In addition, by controlling ambient temperature, L_3_ larval activity was observed at room temperature (20°C) to simulate the quiescent pre-infective stage and at 37°C to mimic an encounter with the human host.

**Fig 1:**
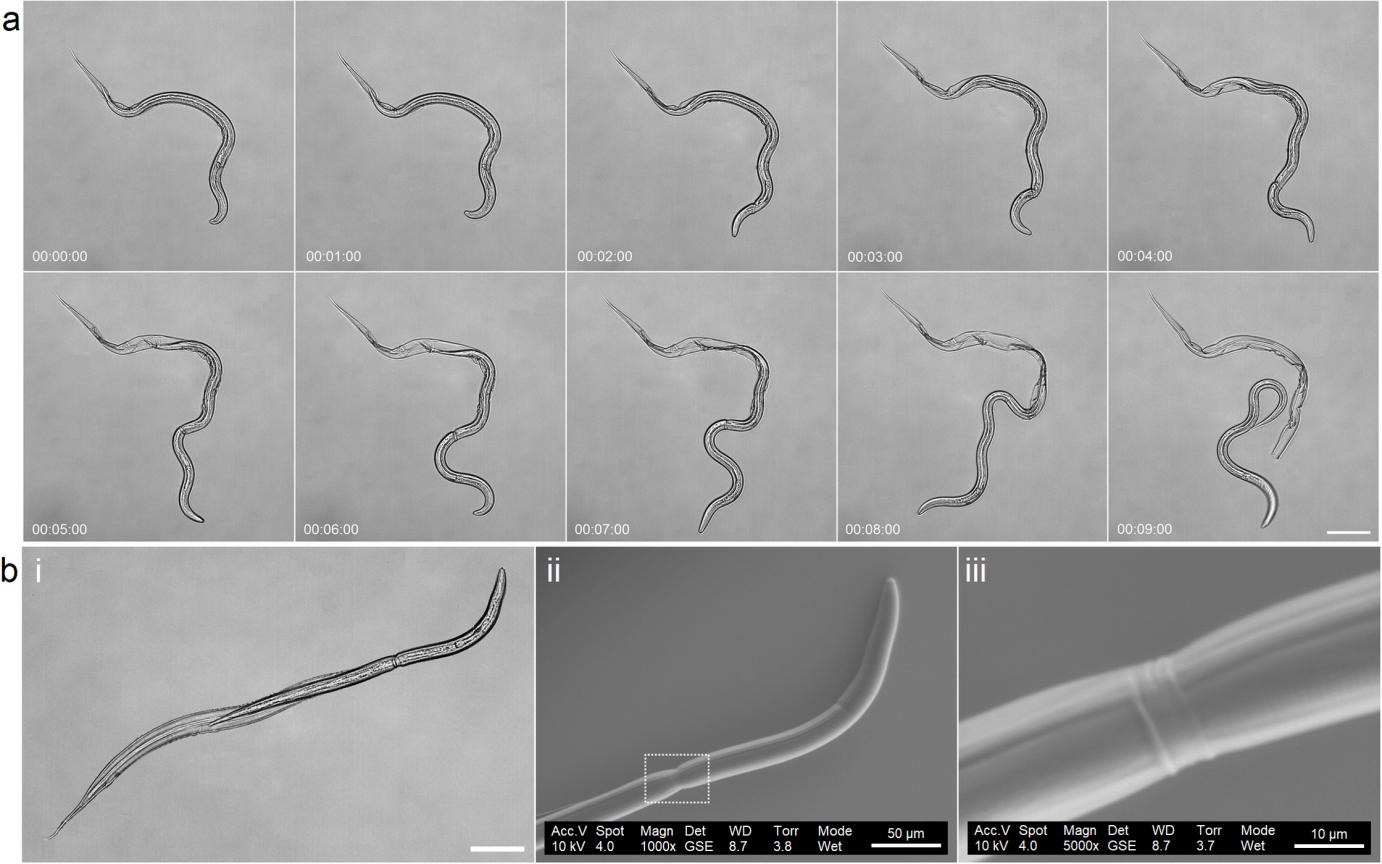
(a) Time-lapse images of L_3_ *N*. *americanus* exsheathing on poly-L-lysine coated glass slides (37°C). After an initial puncture, complete exsheathment occurs within 9 seconds (time is shown as minutes: seconds: milliseconds). Based on our observations *N*. *americanus* L_3_ exsheathment occurs *via* a five-stage process: (I) *Stimulation* (00:00:00)–the *N*. *americanus* demonstrate heightened activity at 37°C, mimicking an encounter with human skin. (II) *Thrashing* (00:00:00–00:01:00)—larva move rapidly and violently. This behaviour expands the sheath in all directions to weaken its integrity and provides greater space for manoeuvre. (III) *Puncture* (00:02:00)—the anterior of the sheath wall is weakened, which can be attributed to the thrashing phase and possibly due to secreted enzymes that have been thought to digest the sheath membrane in its uppermost regions. The larva perforates the anterior sheath tip from which it can emerge. (IV) *Exsheathment* (00:02:00–00:08:00)—The larva manoeuvres out of the punctured sheath, through its characteristic sinusoidal movement, whilst the sheath remains anchored to the surface. The interface of the L_3_ cuticle and sheath is indicated optically by a refractile ring. (V) *Escape* (00:09:00)–The nematode has completely exsheathed and is free to move independently of the sheath. For full movie see S4 Movie and for multiple exsheathments in a single field of view see S1 File and S5 Movie. (bi) Optical and (bii & biii) environmental scanning electron microscopy images for partially exsheathed *N*. *americanus*. Scale Bar (a) = 100 μm, (bi) = 100 μm (bi) 100 μm, (bii) = 50 μm (biii) = 10 μm.

In the absence of poly-L-lysine larvae move freely on glass surfaces. Their activity, as indicated by the rate of sinusoidal movement, is greater at 37°C than 20°C (S1 and S2 Movies, respectively). Even though the ensheathed larvae appear to have enough activity to propel them across the surface of the glass they do not exsheath. In contrast, ensheathed larvae deposited on poly-L-lysine coated slides experience restricted motility. The poly-L-lysine anchors the sheath to the glass surface, such that at 20°C the ensheathed nematode is able only to move within the realms of the static sheath (S3 Movie). At 37°C the poly-L-lysine still provides an anchoring mechanism to attach the sheath to the glass slide; however, the ensheathed larvae are stimulated to lift sections of their anatomy off the poly-L-lysine coated surfaces. The anchoring of the sheath to the glass surface in combination with the increased activity demonstrated at 37°C permits the larvae to exsheath, as the time-lapse images show in Fig 1A (S4 Movie). The exsheathment efficiency was found to be 80.41 ± 1.75% and 1.75 ± 3.04% on poly-L-lysine coated and uncoated surfaces, respectively (see S1 File).

Exsheathed larvae are not anchored to poly-L-lysine and are able to travel across coated surfaces. This movement is irregular, when compared to the controlled sinusoidal movement in the absence of poly-L-lysine, as the exsheathed larvae appear to ‘jolt’ from location-to-location (S5 Movie).

These observations provided an early indication that the physical and chemical properties of the *N*. *americanus* L_3_ cuticle and sheath are different. The interaction between the sheath and poly-L-lysine restricts the movement of ensheathed larvae on coated surfaces. Conversely, the interaction between the exsheathed L_3_ cuticle and the poly-L-lysine is substantially reduced. Therefore, as anionic sites on biological surfaces predominantly bind to cationic poly-L-lysine, these findings suggest the larval sheath could possess a greater abundance of anionic functional groups, with respect to the L_3_ cuticle. Importantly, the use of poly-L-lysine coated glass slides provides a new method for the controlled exsheathment of *N*. *americanus*, such that the sheath and cuticle properties can be studied independently.

### Physical characterisation

Partially exsheathed larval ultrastructure can be captured using chemical or low temperature fixation. Fig 1Bi shows an optical image of a fully hydrated chemically fixed partially exsheathed nematode, with what would appear to be a refractile ring [38–41] present at the interface between the sheath and the L_3_ cuticle. Under environmental scanning electron microscopy (ESEM) the sheath appears to compress the anatomy of the nematode, as indicated in Fig 1Bii and 1Biii. Compression may occur due to differences between the size of the initial sheath puncture, produced by the force generated by the narrow head and the relatively larger width of the emerging anatomy.

By analysing the optical images, the relative size differences between the L_3_ larva and the deposited sheath can be determined. The length and the apparent width (in the middle of the larval anatomy) of the exsheathed L_3_ larva is approximately 542 ± 25 μm and 25 ± 3 μm, respectively. In contrast, the sheath length and apparent width (at the centre of the sheath measured after the exsheathment process) is approximately 610 ± 28 μm and 38 ± 2 μm, respectively. These findings indicate the length and the apparent width of the sheath is greater than the emergent L_3_ larva. It is important to note that the apparent width does not account for the three-dimensional properties of the emergent L_3_ larva or sheath. Therefore, the circumference, which can be calculated by determining the height of the three-dimensional structures, provides an improved description of the L_3_ larva and sheath physical properties.

Accurate physical characterisation of the emergent larva and sheath height, as well as QNM information such as adhesion forces, can be determined using AFM [42]. The height of exsheathed *N*. *americanus* and double sheath layer were 4.26 ±0.20 μm and 0.71 ± 0.04 μm respectively, such that their circumferences can be estimated to be ~56 μm and ~76 μm, respectively. Therefore, the thickness of the sheath, which acts as a protective hydrating shield, is extremely thin at 0.35 μm.

**Fig 2:**
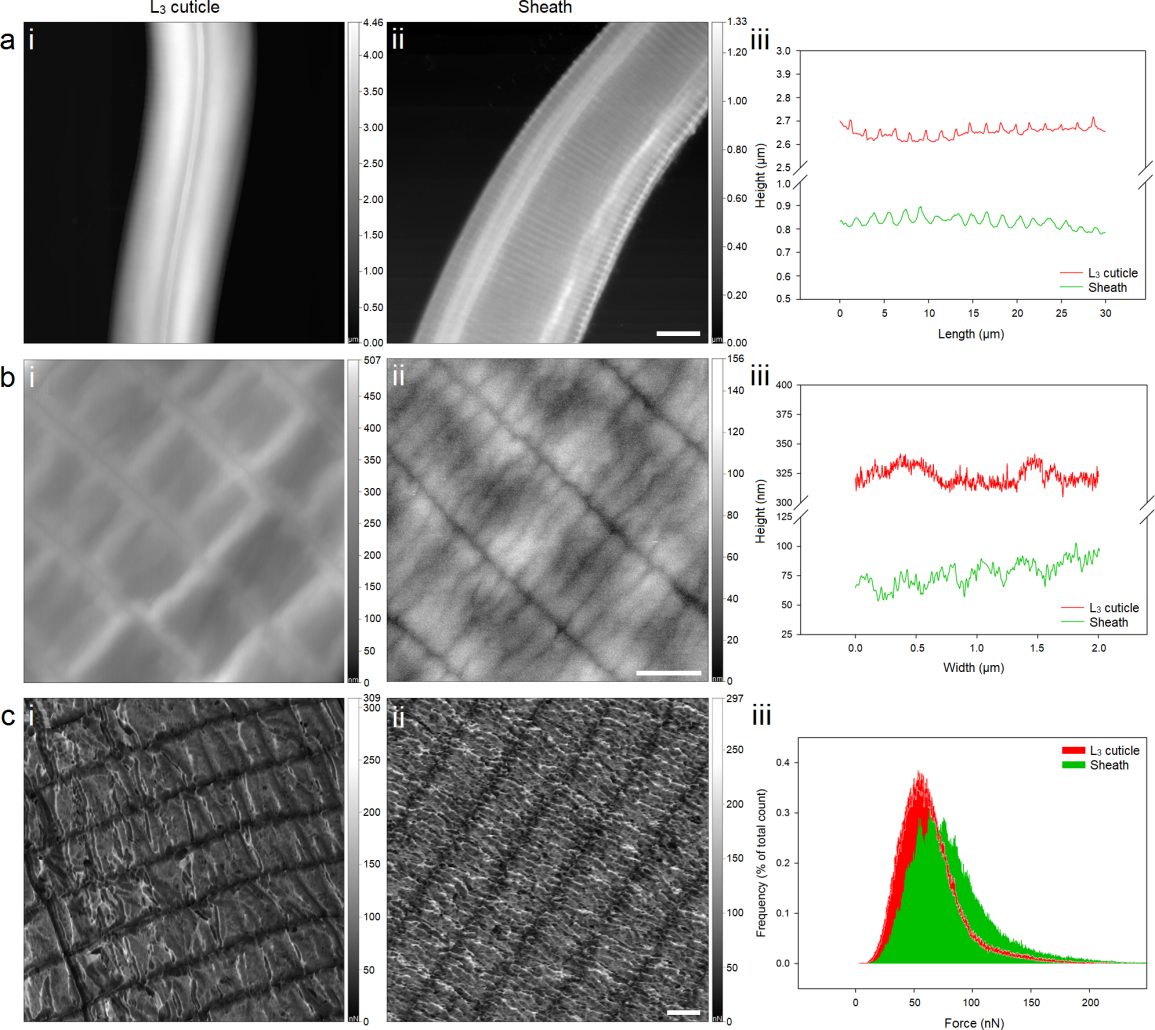
(a) Low and (b) high resolution AFM topographical images of (ai & bi) exsheathed larva and (aii & bii) sheath surface with corresponding (aiii) length and (biii) width data. (aiii) The length of the nematode anatomy exhibits repeating annuli on the L_3_ cuticle and sheath, which are separated by 1.72 ± 0.13 μm and 1.80 ± 0.25 μm, respectively. (biii) The sheath also exhibits nano-annuli, which are found in-between and perpendicular to the annuli, and occur every 323 ± 18 nm, with depths between nano-annuli of 29 ± 5 nm. Adhesion force images for (ci) cuticle and (cii) sheath measured using AFM tip functionalised with poly-L-lysine. (ciii) The histogram shows the differential distribution of adhesion force between poly-L-lysine functionalised tip and L_3_ cuticle and sheath surface. Scale bar for a = 10 μm, b = 1 μm and c = 1 μm.

Analysis of the surface topography revealed both cuticle and sheath possess primary structure, in the form of repeating surface annuli, Fig 2Ai & 2Aii. Annuli on the cuticle are closer together and shallower, occurring every 1.72 ± 0.13 μm with depths between annuli of 35 ± 12 nm, when compared to the sheath, which occur every 1.80 ± 0.25 μm with depths between annuli of 42 ± 15 nm, Fig 2Aiii. These differences between the spacing of the annuli can be attributed to the relative maturity of the sheath to the growing L_3_ larva, and the exsheathment process, which is known to expand the sheath in all directions.

Additionally, the sheath exhibits a secondary structure, nano-annuli, which are found in-between and perpendicular to the annuli, Fig 2Bii. The periodicity and depth of the nano-annuli found on the sheath surface is 323 ± 18 nm and 29 ± 5 nm. This additional surface topography enhances the sheaths surface area by ~ 4%, when compared the L_3_ cuticle (see S1 File). This surface area enhancement may contribute to the differential adherence observed for ensheathed L_3_ larva as well its exsheathment on solid supports and during infection. However, due to the marginal degree of surface area enhancement we postulate the surface topography of the sheath may play a minor, although important, role in its overall infection mechanism.

Determination of the adhesion forces between coated surfaces and the larval cuticle and sheath permit quantification of our observations that show ensheathed larva exhibit restricted motility when in contact with poly-L-Lysine. Images for the adhesion forces measured between poly-L-lysine functionalised tip and cuticle and poly-L-lysine functionalised tip and sheath are shown in Fig 2Ci and 2Cii, respectively. Fig 2Ciii shows the distribution of the measured adhesion forces between the functionalised tip and sheath were 33 ± 6% greater than the adhesion forces between a functionalised tip and the cuticle. These measurements are in agreement with our observations of limited motility of ensheathed *N*. *americanus* on poly-L-lysine coated glass surfaces in comparison with exsheathed L_3_ larvae. These results suggest there are differences in the surface chemistries of the sheath and cuticle, which were explored further with ToF-SIMS.

### Chemical characterisation

From the outset it is important to note L_3_ larva and their sheaths are complex biochemical structures and are challenging to analyse and interpret.

ToF-SIMS was used to directly probe the cuticle and sheath surface chemistry. Partially exsheathed *N*. *americanus* samples were prepared (n = 12) to obtain surface chemical data from the cuticle and sheath. Data analysis of these surfaces was approached in a systematic manner using objective processes, specifically PCA analysis, MCR analysis and statistical scoring of the full data series (as shown in the S1 File).

**Fig 3:**
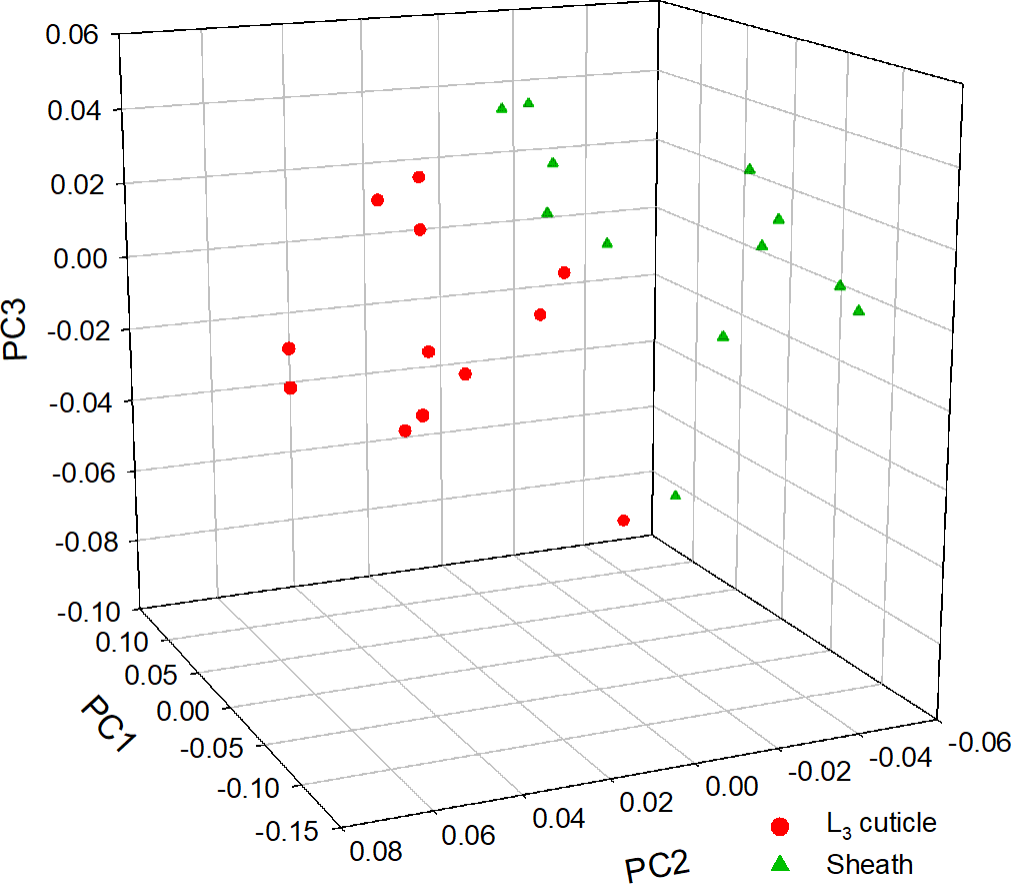
Three-dimensional principal component scores plot for PC 1, 2 and 3 for the ToF-SIMS spectra (no. m/z peaks = 822) of partially exsheathed L_3_ *N*. *americanus* in both negative (no. m/z peaks = 429) and positive polarity (no. m/z peaks = 393). PCA analysis was conducted on 24 different regions of interest (cuticle = 12, sheath = 12).

PCA analysis was conducted on the full data series of 12 partially exsheathed axenic *N*. *americanus* using regions of interest to distinguish the cuticle from sheath. Preliminary analysis of the normalised eigenvalues on the first ten principal components shows PC 1 (59.6%), 2 (18.9%) and 3 (12.0%) account for 90.5% of the variability within the data set (see S1 File). When a three-dimensional scores plot is generated for the first three principal components the data unambiguously separates into two distinct populations, Fig 3. However, although the PCA analysis appears to separate the sheath and cuticle, due to the complexity of the dataset under consideration 95% confidence limits on individual PC loadings alone were not able to identify mass ions that were significantly expressed on cuticle or sheath surface. This observation can be attributed to the high degree of biological variability within the data series. Therefore, in order to reduce the variability, surface chemical differences within a data set (single partially exsheathed larva) were investigated using MCR image analysis.

**Fig 4:**
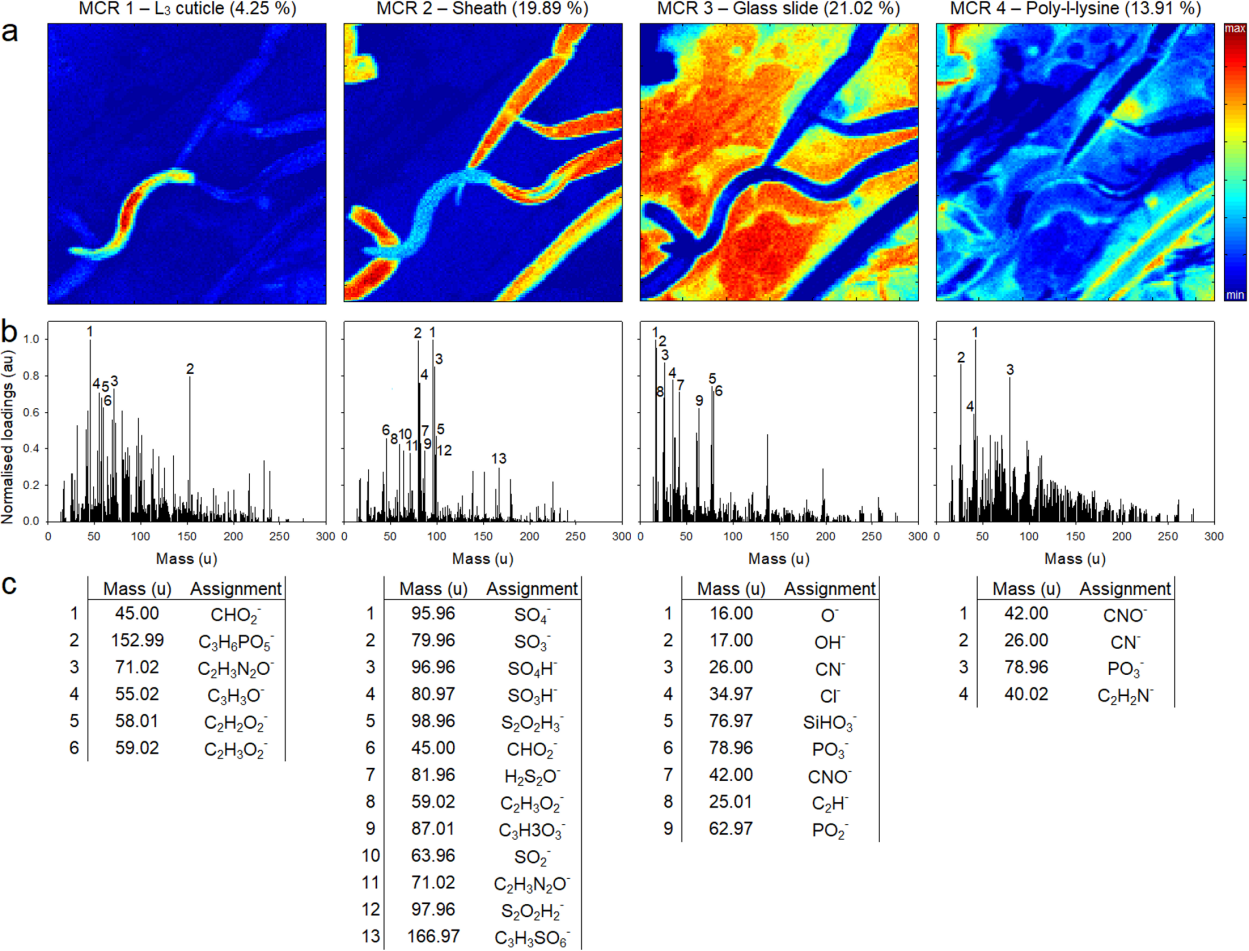
(a) False colour score heat maps (b) normalised loadings and (c) chemical identities of highly loaded mass ions for MCR components 1 to 4. For residual scores, loadings and chemical identities of highly loaded mass ions, see S1 File. Scale bar = 50 μm.

On first inspection the MCR scores heat maps, Fig 4A, were able to differentiate the different chemical surfaces on display, such that MCR components 1, 2 and 3 are specific for L_3_ cuticle, sheath and glass substrate, respectively. MCR component 4 demonstrates non-specificity and may indicate the presence of poly-L-lysine residues. Through analysis of the highly loaded mass ions for MCR components 1–4 (Fig 4B), chemical identities were assigned (Fig 4C). A detailed table of mass assignment interpretations of MCR 3, 4 and MCR residuals, and reconstructed images of highly loaded ions for MCR 1–4 are provided in the S1 File.

#### Cuticle

**Fig 5:**
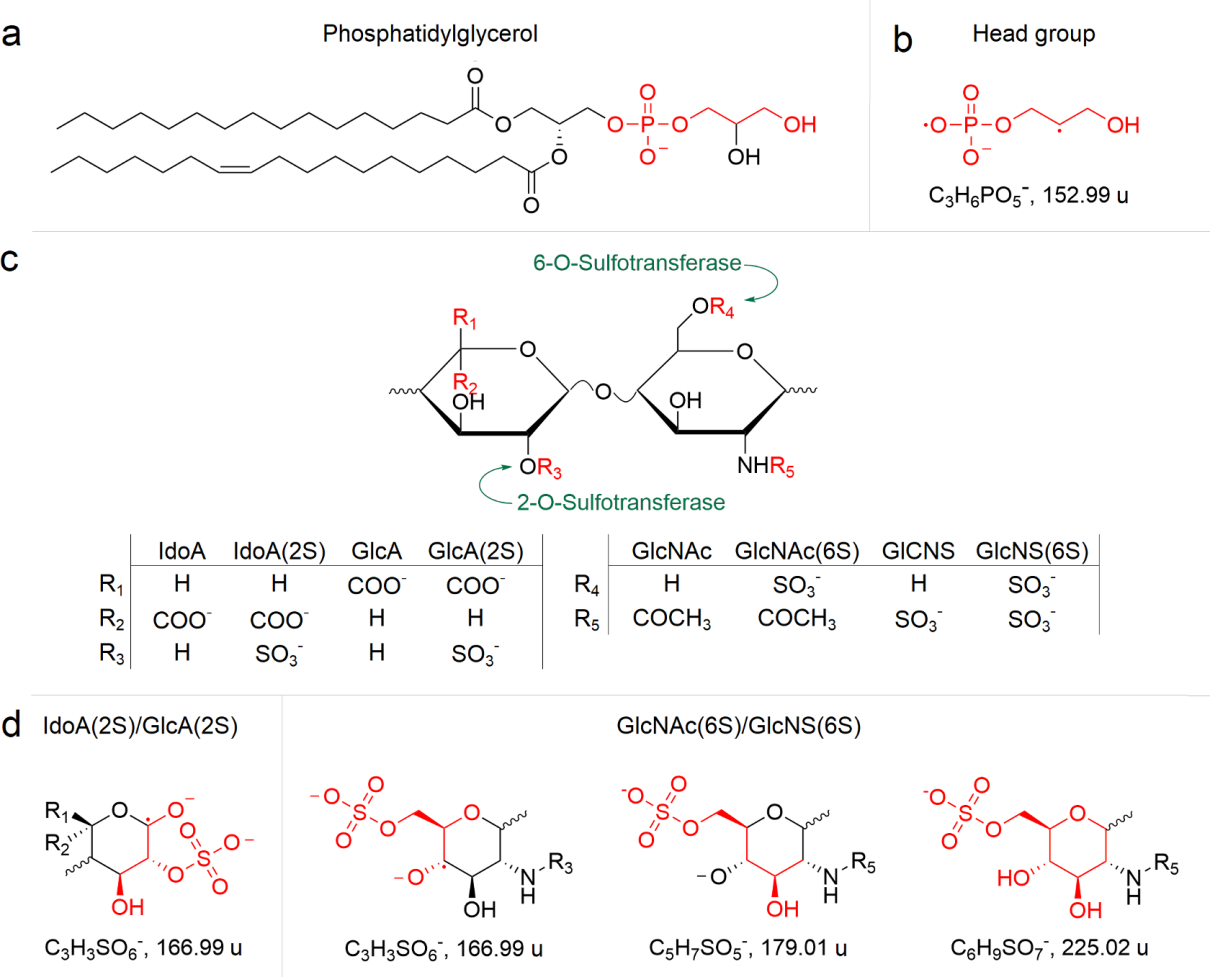
(a) Phosphatidylglycerol and (b) corresponding head group fragment. (c) Heparan sulphate disaccharide unit composed of uronic acid (left) and glucosamine (right) monosaccharide subunits. (d) Heparan sulphate monosaccharides, with identified chemical fragments in highlighted in red. Sulphation of the disaccharide subunit is conducted by heparan sulphate 2-O-sulfotransferase and heparan sulphate 6-O-sulfotransferase.

The loadings for MCR component 1 possess two highly loaded mass ions at 45.00 u and 152.99 u, identified as the CHO_2_^-^ and a C_3_H_6_PO_5_^-^ ion. The CHO_2_^-^secondary ion, a formic acid functional group, is distributed over both cuticle and sheath; however, it is located in greater abundance on the cuticle. Through further analysis of the highly loaded ions elucidates that methoxide ions, CH_3_O^-^ (31.02 u), are also specific to the cuticle, whereas the C_2_H_3_O^-^ (43.02 u), CHO_2_^-^ (45.00 u) C_3_H_3_O^-^ (55.02 u), C_2_H_2_O_2_^-^ (58.01 u), C_2_HO_3_^-^ (72.99 u), C_2_H_2_N_2_O^-^ (83.02 u), C_3_H_2_O_3_^-^ (86.00 u), C_4_H_7_NO_2_^-^ (101.4 u) and C_5_H_7_NO_2_^-^ (113.05 u) ions exhibit greater expression on L_3_ cuticle surfaces. Of particular interest is the C_3_H_6_PO_5_^-^ ion, which indicates a phosphatidylglycerol head group, and is located specifically on the exposed L_3_ cuticle surface and has been described as a diagnostic fragment of phosphorylated lipids, Fig 5A and 5B [43].

The structural [44] and functional [45] role of membrane bound lipid molecules, such as phosphatidylglycerol, are yet to be determined in great detail. Nevertheless, they are thought to lubricate biological surfaces as well as regulate the permeability of cell membranes and the posttranslational modification of other membrane bound molecules [46].

#### Sheath

As shown in Fig 4C the highly loaded mass ions for MCR component 2 (associated with the sheath) appear to contain sulphated compounds. Analysis of their distribution suggests they are found on both cuticle and sheath surfaces, although they are located in greater abundance on the sheath. This observation is also true for the C_2_H_3_O_2_^-^, C_3_H_3_O_2_^-^ and C_3_H_3_O_3_^-^ ions, whereas the formic acid functional group denoted by the CHO_2_^-^ ion, demonstrates greater abundance on the cuticle when compared to the sheath. Further analysis of the highly loaded compounds for MCR component 2 (see S1 File), reveals a group of structurally related sulfonate compounds with low mass deviation, including C_2_H_3_SO_5_^-^ (138.98 u, 50.76 ppm), C_3_H_3_SO_5_^-^ (150.98 u, 41.89 ppm), C_3_H_3_SO_6_^-^ (166.97 u, 41.92 ppm), C_5_H_7_SO_5_^-^ (179.01 u, 28.60 ppm) and C_6_H_9_SO_7_^-^ (225.02 u, 64.56 ppm) ions. Image reconstruction for the structurally related sulphated mass ions indicates the secondary ions at 166.97 u, 179.01 u and 225.02 u are found specifically on the larval sheath. These findings conform to the primary observation that MCR component 2 is highly loaded with sulphur containing compounds.

The secondary ions specifically found on the sheath surface exhibit similarities to heparan sulphate like monosaccharides [47]. Heparan sulphates are highly negatively charged polysaccharides, composed of alternating uronic acid (iduronic acid (IdoA) or glucuronic acid (GlcA)) and glucosamine subunits (*N*-acetylglucosamine (GlcNAc) or *N*-sulphoglucosamine (GlcNS)) that are usually found bound to core extracellular matrix and cuticle proteins, such as collagen [48]. Nematode cuticles undergo extensive post translational modification [49], such as sulphation [50], during their development from larvae to adults. Examination of the *N*. *americanus* genome [51] indicates there are genes encoding for heparan sulphate 2-O-sulfotransferase and heparan sulphate 6-O-sulfotransferase. Heparan sulphate 2-O-sulfotransferase is responsible for the sulphation of GlcA to GlcA(2S) and IdoA to IdoA(2S) [52], whereas heparan sulphate-6-sulfotransferase is responsible for the sulphation of GlcNAc to GlcNAc(6S) and GlcNS to GlcNS(6S), [53] Fig 5C [54]. Based on these findings and the ability of ToF-SIMS to observe monosaccharide subunits, taking into account its destructive nature when producing secondary ions, we propose the surface of larval sheath is likely to contain heparan sulphate disaccharide subunits IdoA(2S)/GlcA(2S)-(1–4)-GlcNAc(6S)/GlcNS(6S), Fig 5D.

Heparan sulphates contribute to a variety of physical and biochemical activities [55] and have been implicated in many biological functions [56], which include enhanced host infection and colonisation by parasites, *e*.*g*. the attachment and cellular invasion of *Toxoplasma gondii* [57], and immune modulation, *e*.*g*. chemokine presentation and recruitment of lymphocytes and dendritic cells [58]. Therefore, we postulate the presence of heparan sulphate like monosaccharides on the sheath surface can be explained by its relative maturity, when compared to the immature cuticle. This key difference could help explain why the cuticle and sheath demonstrate differential binding to cationic poly-L-lysine coated surfaces, due to the adherence of highly anionic heparan sulphate like monosaccharides. Furthermore, when considering heparan sulphate, an immunologically active molecule, this could provide rationale as to how the exsheathed larvae are able to evade host immune defences, through deposition of a diversionary immune modulatory heparan sulphate enriched sheath nidus.

**Fig 6:**
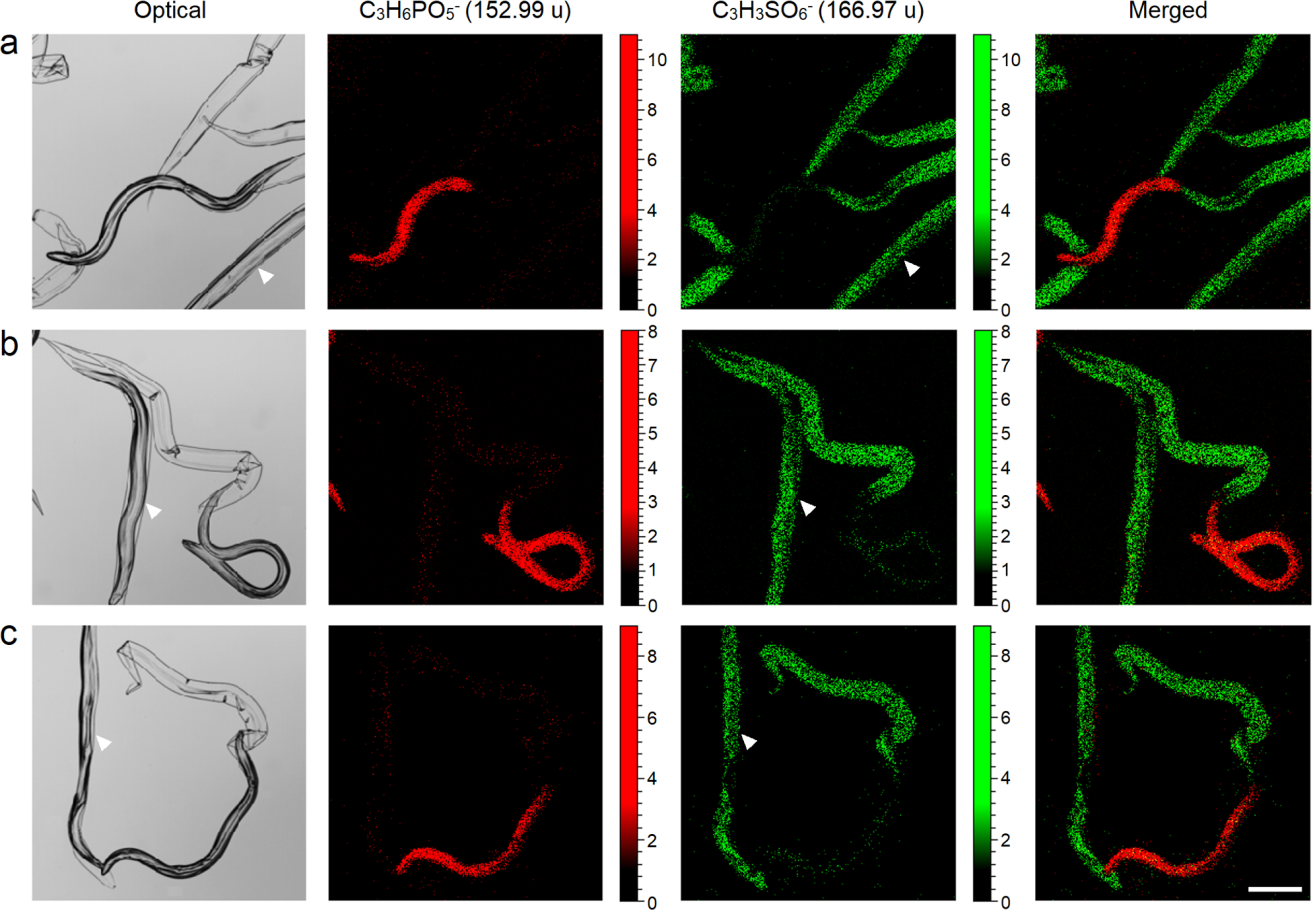
Comparative ion intensity images and spectra showing chemical differences between the L_3_ cuticle and sheath for three different (a, b & c) partially exsheathed larvae, where the optical image in Fig 6a corresponds to the MCR data shown in Fig 4A. Arrow heads indicate ensheathed larvae that can be visualised optically but cannot be distinguished using chemical image reconstruction. Scale bar = 100 μm.

Fig 6 shows optical and secondary ion images for three different partially exsheathed *N*. *americanus* and their corresponding cuticles and sheath, using mass ions 152.99 u and 166.97 u, respectively (p<0.01, n = 12). The optical images demonstrate how light microscopy is able to visualise exsheathed larvae, sheaths and ensheathed larvae (arrow heads). The secondary ion images clearly show the differences between exsheathed larvae and sheaths, however, as with many surface analysis techniques ToF-SIMS is unable to highlight the ensheathed larvae without depth profiling [59]. The immune system is analogous to surface analysis techniques and is unable to identify antigenic species that are hidden behind physical barriers. Therefore, Fig 6 provides a visual representation to how the body may perceive different biological surfaces, such that ensheathed larvae are temporarily chemically invisible to the host’s immune defences due to the physical barrier presented by the sheath. We postulate this effect could be enhanced by the potentially antigenic heparan sulphate like molecules present on the sheath surface, which may act as an immunological sink and divert the host immune defences away from exsheathed larvae.

The methods we have developed to separate the larval sheath from cuticle and the differential chemistries that have been identified on different hookworm surfaces paves the way for an in depth characterisation of cellular interactions with larval surfaces [60], whilst simultaneously investigating the relative immunogenicity of each surface [61].

### Conclusion

An investigation of the physical and chemical properties of the emerging infective L_3_ *N*. *americanus* larvae has been performed in unprecedented detail. Differences have been demonstrated between larval sheath and cuticle that may provide an insight into the biology of their percutaneous infections. A method to control the exsheathment process has been established, which enables simple separation of the *N*. *americanus* sheath from cuticle. AFM and ToF-SIMS were used to analyse of the surface properties of resulting cuticles and sheaths. AFM revealed the sheath possesses surface area enhancing secondary structure (nano-annuli), which are found in-between and perpendicular to the annuli. Additionally, QNM demonstrated greater adhesion forces between a cationic functionalised tip and sheath than tip and cuticle confirming the observations of limited *N*. *americanus* motility on poly-L-lysine coated surfaces. The application of ToF-SIMS and multivariate analysis revealed significantly different chemistries on the cuticle and the sheath. Specifically, the cuticle expressed phosphatidylglycerol head groups, whereas the sheath exhibited the presence of heparan sulphate-like monosaccharides. We postulate the anionic heparan sulphate-like monosaccharides facilitated sheath adherence to the cationic poly-L-lysine coated solid supports, a phenomenon which may be replicated *in vivo*. Furthermore, the differential chemistries discovered may have an influence on immune evasion as well as successfully avoiding host immunological barriers *via* deposition of an immunologically active sheath. We believe the development and application of this analytical approach enables the direct probing of innate parasite surfaces, which will enhance understanding of infection processes.

## Materials and methods

### Materials

#### Poly-L-Lysine coated slides

Methanol (HPLC grade), acetone (HPLC grade) and microscopy glass slides (76 x 26 x 1.0–1.2 mm), were purchased from Thermo Fisher Scientific (Hemel Hempstead, United Kingdom). Poly-L-lysine (70–150 kDa) was purchased from Sigma-Aldrich (Gillingham, United Kingdom). Argon gas was acquired from BOC Gasses (Manchester, United Kingdom).

#### N. americanus

*N*. *americanus* was sourced from Papua New Guinea [62, 63]. L_3_ larvae were prepared under a licence from the Medicines and Healthcare Products Regulatory Agency (MHRA MIA IMP 3057). The microbial bio-burden of the larvae was assessed by an MHRA approved contractor (FDAS, BioCity, Nottingham). All L_3_ larvae used in the current study were deemed to be axenised, in that they were free from gram negative and positive bacteria, and fungi.

### Methods

#### Poly-L-lysine coated slides

Ultrapure deionised water, methanol, acetone and argon were used to clean and dry microscopy glass slides. A solution of poly-L-lysine in deionised water (0.01%, 70–250 kDa, 100 μL) was pipetted at the centre of the slide. Another clean microscopy slide was placed on top the poly-L-lysine to distribute the poly-L-lysine uniformly over the surfaces of both slides. After a 5 minute incubation the slides were carefully separated. Ultrapure deionised water was used to wash and remove excess poly-L-lysine and argon was used to dry the slides. Water contact angle (WCA) was used to confirm the presence or absence of polymer coat (S1 File).

#### Preparation of *N*. *americanus* slides

*N*. *americanus* were washed with sterile ultra-pure deionised water (15 ml, 5 times), using vortexing and centrifugation (1500 rpm, 1 min). The *N*. *americanus* (n>500) suspensions were aspirated to 2 mL and pipetted on to poly-L-lysine coated microscopy slides. The larvae were permitted to settle on to the glass and anchor to the poly-L-lysine coated surface. The slides were placed in a temperature controlled incubator (37°C, ibidi Heating & Incubation System) to encourage exsheathment. To ensure the preparation of samples containing partially exsheathed hookworms, slides were screened using optical microscopy. Preservation of partial exsheathment was achieved by drying the slides with argon, to remove excess water, followed by immediate storage in an air tight sealed container at -20°C, to prevent further dehydration.

#### Optical microscopy

Exsheathment was optically imaged using an inverted Nikon Eclipse TE 300 microscope, equipped with a Nikon Plan Flour 4x 0.13 NA, 10x 0.30 NA and 20x 0.50 NA air objectives, CoolLED pE-4000 light source, QImaging optiMOS sCMOS camera (1920 x 100, 100 fps) and open source Micromanager software (version 1.4). S1–S5 Movies demonstrating the effects of poly-L-lysine (±) and temperature (37°C and 20°C) on larval exsheathment were captured simultaneously using suspensions from the same batch of *N*. *americanus* larva for all conditions (larvae per condition > 20, experimental batches and repeats = 3). L_3_ larva length and diameter and sheath width and length were determined using open source Fiji software (L_3_ larvae n = 50 and sheaths n = 50).

#### Environmental Scanning Electron Microscopy (ESEM)

Axenic L_3_ larvae were prepared and exsheathed on poly-L-lysine coated slides using the methods described above. To preserve the integrity of *N*. *americanus* structural architecture during ESEM imaging, partially exsheathed adhered nematodes were fixed (glutaraldehyde 3% v/v, 12 hours), rinsed (ultrapure deionised water 50ml, 3 times) and partially sublimated, to remove surface water, under ESEM vacuum. Samples were imaged using a Philips XL30 ESEM-FEG environmental scanning electron microscope (20 kV, 10.1 mm working distance).

#### Atomic Force Microscopy (AFM)

Prior to imaging microscopy slides containing sheathed, partially exsheathed and exsheathed L_3_ larva were equilibrated to room temperature to remove surface water, acquired during refrigeration. To prevent movement during imaging slides were fixed to the AFM stage using adhesive tape. Samples were imaged with a Bruker Dimension FastScan AFM, using a Bruker cantilever (RTESPA-150, resonant frequency 150 kHz, spring constant 6 N/m and tip radius 8 nm), and FastScan Icon head. *N*. *americanus* L_3_ larva, partially exposed cuticle and sheathes were imaged using scan sizes of 75x75 μm, 10x10 μm and 5x5 μm at 1024 force curves/line and scan rate of 0.5 Hz (n = 3). It is important to note the difference in image features between the AFM images for the worm, Fig 2Ai, and sheath, Fig 2Aii, is due to the 6-fold difference in height. Bearing this in mind the data shown for Fig 2Aiii has been extracted from comparable regions of interest that overcome height differences. Adhesion measurements were conducted using tips functionalised with poly-L-lysine (0.01% w/v, 5 minutes, followed by ultra-pure deionised water rinsing) on comparable regions of interest (10 μm^2^, n = 3). Data was processed and analysed using open source Gwyddion software (version 2.41).

#### Time-of-flight Secondary Ion Mass Spectrometry (ToF-SIMS)

ToF-SIMS produces hyperspectral data sets, such that each different pixel on a reconstructed image corresponds to a unique mass spectrum. Therefore, to identify the heterogeneities within this ‘information rich’ dataset, in particular the masses that differentiate L_3_ cuticle from its sheath, MCR analysis was applied. MCR analysis deconstructs hyperspectral datasets without prior knowledge of its composition using an alternating least squares (ALS) algorithm [64]. Through analysis of the MCR scores and loading plots, for the data generated using negative polarity ToF-SIMS, the spatial coordinates that demonstrate chemical similarity were grouped together and the mass ions that correspond to key differences were identified, respectively. Accurate mass lookup tables were used to decipher the chemical identities of highly loaded mass ions.

Slides containing immobilised *N*. *americanus* were transferred directly from refrigeration to the ToF-SIMS instrument. The samples and vacuum were permitted to equilibrate overnight prior to data acquisition. Negative and positive polarity data were recorded using a ToF-SIMS IV instrument (ION-TOF GmbH, Münster, Germany) equipped with Bi_3_^+^ primary ion source (25 kV), and a single-stage reflectron analyser. A flood gun producing low energy electrons (20 eV) was employed to compensate for surface charging caused by the positively charged primary ion beam on the charge insulating sample surface. To ensure static conditions the total primary ion beam did not exceed 1×10^−12^ ions.cm^-2^. Regions of interest (500 × 500 μm) were captured at a resolution of 256 × 256 pixels. Secondary ion mass spectra were collated over the mass range of 0–1000 amu in both positive and negative polarity for a total of separate 12 partially exsheathed larvae.

#### Multivariate analysis of hyperspectral datasets

Hyperspectral datasets obtained from the ToF-SIMS were analysed with PCA analysis and MCR analysis (PLS_Toolbox (5.2), Eigenvector Research).

Principal Component analysis was conducted on partially exsheathed axenic *N*. *americanus* (n = 12) captured using low temperature fixation, to maintain structural integrity, whilst omitting chemical interferents. Regions of interest (ROI) were selected from the apex of the L_3_ cuticle and sheath, so that the differential surfaces can be distinguished from each other and to maximise the mass resolution whilst minimising measurement artefacts that could be generated from the topographical features present on hookworm surfaces. A m/z peak list was generated for the negative ion polarity by applying an automated search to the mass spectra for L_3_ cuticle and sheath pairs. The peak lists were later combined and applied to mass spectra for all 12 partially exsheathed nematodes. Topographical features, such as irregular or rounded surfaces can generate measurement artefacts. Therefore to facilitate comparison the ion counts for each m/z peak were normalised to the ROI total ion count, which has been shown to reduce topographical deviations [65]. The process was repeated for the positive ion polarity. The data was assembled into a signal spreadsheet composed of ROIs selected from the apex of 12 different L_3_ cuticles and 12 different sheathes (total ROIs = 24), and their corresponding normalised m/z ion counts for m/z values up to 665 m/z and 281 m/z for negative and positive polarities, respectively. The total number of m/z peaks investigated was 822 (429 negative m/z peaks and 393 m/z peaks). The data obtained from the mass spectra were systematically studied to determine whether the L_3_ cuticle and sheath cold be chemically differentiated, by: 1) calculating the correlation coefficients for the 12 different L_3_ cuticles and sheath pairs, 2) performing principal component analysis on 24 different ROIs (12 L_3_ cuticles and 12 sheaths), 3) application of Student’s t-test and data filters to categorise masses for L_3_ cuticle or sheath surface specificity and 4) reconstructing ToF-SIMS images and implementing image contrast analysis to visually identify indicators of L_3_ cuticle and sheath chemical disparity.

MCR analysis was conducted using PLS_Toolbox (5.2), (Eigenvector Research). The main article of this publication focuses on the data obtained from a representative partially exsheathed *N*. *americanus* L_3_ larva using negative mode ToF-SIMS, due to its ability to successfully distinguish different surface chemistries. For positive mode MCR analysis see S1 File. MCR analysis was conducted using residual four components, which was determined using principal component analysis (PCA) pre-screen by ascertaining the number of principal components that account for distinct variability within the data set. Scores images and component loadings were reconstructed using MatLab (R2013b) and SigmaPlot (11.0). Mass assignments were made using accurate mass lookup tables (IONTOF (6.5)), for which possible structures were identified and reconstructed using ChemSpider (http://www.chemspider.com) and ChemBioDraw Ultra (13.0). MCR analysis was supported by correlation coefficient analysis, PCA (MatLab (R2013b)) and statistical scoring between data set of 12 different partially exsheathed nematodes with defined regions of interest for L_3_ cuticle (n = 12) and sheath (n = 12) (see S1 File).

## Supporting information

S1 File This data file contains supporting methods for water contact angle (WCA) and exsheathment efficiency. In addition, this file provides results and detailed analysis for supplementary WCA, AFM, ToF-SIMS, PCA, MCR, and statistical scoring data.
(PDF)

S1 Movie Exsheathment behaviour of *N*. *americanus* at 37°C in the absence of poly-l-lysine coated glass substrate. (MP4)

S2 Movie Exsheathment behaviour of *N*. *americanus* at 20°C in the absence of poly-l-lysine coated glass substrate. (MP4)

S3 Movie Exsheathment behaviour of *N*. *americanus* at 20°C in the presence of poly-L-lysine coated glass substrate. This movie was captured using an inverted optical microscope and shows a mobile larva on top of stationary larva. The mobile larva is able to move the centre of its anatomy, because it is only in partial in contact with the poly-L-lysine surfaces at the head and tail. Whereas full the length of stationary larva is in contact with the coated surface and as a result its movement is restricted.
(MP4)

S4 Movie Exsheathment behaviour of *N*. *americanus* at 37°C in the presence of poly-L-lysine coated glass substrate (single larval exsheathment). (MP4)

S5 Movie Exsheathment behaviours of *N*. *americanus* at 37°C in the presence of poly-L-lysine coated glass substrate (mass exsheathment). (MP4)
