# Correction: The physicochemical fingerprint of *Necator americanus*

**DOI:** 10.1371/journal.pntd.0010531

**Published:** 2022-06-08

**Authors:** Veeren M. Chauhan, David J. Scurr, Thomas Christie, Gary Telford, Jonathan W. Aylott, David I. Pritchard

Reference 42 is cited incorrectly. The correct citation is: Fakhrullina G, Akhatova F, Kibardina M, Fokin D, Fakhrullin R. Nanoscale imaging and characterisation of *Caenorhabditis elegans* epicuticle using atomic force microscopy. Nanomedicine: Biology, Medicine and Nanotechnology. 2017;13(2):483–491. pmid: 27771431
