# Supplementary material for: The physicochemical fingerprint of Necator americanus
Source: PLoS Negl Trop Dis. 2017 Dec 7;11(12):e0005971. doi: 10.1371/journal.pntd.0005971 (PMC5720516; doi:10.1371/journal.pntd.0005971)
Supplement: S1 File — In addition, this file provides results and detailed analysis for supplementary WCA, AFM, ToF-SIMS, PCA, MCR, and statistical scoring data. (PDF) [file pntd.0005971.s001.pdf]

Supporting Information

The Physicochemical Fingerprint of *Necator Americanus*

Veeren M Chauhan*, David J Scurr, Thomas Christie, Gary Telford,
Jonathan W Aylott*, David I Pritchard*

School of Pharmacy, Boots Science Building, University of Nottingham, Nottingham, United Kingdom

***Corresponding Authors:** Veeren.Chauhan@nottingham.ac.uk (VMC);

Jon.Aylott@nottingham.ac.uk (JWA); David.Pritchard@nottingham.ac.uk (DIP)

This PDF file includes:

Supporting Methods

Supporting Results

Figures S1 -S17

Tables S1 to S3

Captions for Movies S1 to S5

Other Supporting Information for this manuscript includes the following:

Movies S1 to S5

Materials and Methods

Water contact angle

WCA was used to confirm the presence or absence of polymer coat. WC measurements were determined using a CAM 200 optical contact angle meter (KSV instruments Ltd) equipped with Pendant Drop Surface Tension Software (Version 3.42). Poly-L-lysine treated and untreated glass slides returned WCA's of 67.27 ± 5.33° (Figure S1A) and 5.15 ± 1.09° (Figure S1B), respectively (n=3).

Exsheathment Efficiency

The efficiency of larval exsheathment was determined by depositing ensheathed larva (n=20) suspended in deionised water on the surface of either poly-L-lysine coated and uncoated glass surfaces (n=3), which were incubated at 37 °C for 30 minutes. The number of exsheathed larva, ensheathed larva and sheaths were counted after incubation from which a percentage of exsheathment was calculated (Figure S1C).

Results

Atomic Force Microscopy

The nano-annuli provide the sheath with an enhanced surface area, when compared to the L_3 cuticle. The surface area of the sheath can be approximated through calculation of the surface area for a single nano-annulus, using the elliptical cylinder surface area approximation equation.

$$Surface\ area\ of\ nano-annuli \approx l\frac{\pi[3(r-d)-\sqrt{(3r+d)(r+3d)}]}{2}$$

where radius (*r*) = 0.162 µm, depth (*d*) = 0.029 µm and length (*l*) = 1.800 µm. Therefore the surface area of a single nano-annulus is ~ 0.603 μm^2, such that for field of view measuring 10x10 µm, the surface area for an L_3 cuticle and sheath is ~100 μm^2 and ~104 μm^2, respectively, Figure S2.

Principal component analysis (PCA)

Primarily, PCA of the complete dataset (12 different larva & their corresponding 12 sheaths) was applied to determine if the surfaces are statistically different and can be differentiated from each other. Preliminary analysis of the normalised eigenvalues on the first ten principal components shows PC 1 (59.6 %), 2 (18.9 %) and 3 (12.0 %) account for 90.5 % of the variability within the data set Figure S4. In depth analysis of the principal component scores confirms the complexity of the biological surfaces under investigation as no component alone separate the L_3 cuticle and sheath surfaces in the vertical axis, Figure S5 A-C. Although, PC2 and PC3 show scores for L_3 cuticle are always greater in PC2 and lower for PC3, when compared to the sheath. Analysis of component pairs PC1 & PC2, PC1 & PC3, and PC2 & PC3 show L_3 cuticle and sheath cluster into discrete groups with marginal overlap between populations, Figure S5 D-E. Specifically, the component pair of PC2 & PC3 exhibits the greatest separation. When a three dimensional scores plot is generated for the first three principal components the data unambiguously separates into two distinct populations.

Secondly, through detailed analysis of the PCA loadings, calculation of the significant differences between the L_3 cuticle and sheath, application of data filters and image analysis mass ions that are

unique or were significantly expressed on each surface were identified. Through examination of both mean centred and auto-scaled loadings for the first three principal components it was established PC2 and PC3 correspond to markers for the sheath and L_3 cuticle, Figure S6. Specifically, the sheath could be identified by low and high loadings on PC2 and PC3, respectively, whereas the L_3 cuticle could be identified by high and low loadings on PC2 and PC3, respectively. However, due to the complexity of the dataset under consideration 95% confidence limits on PC loadings alone were not able to identify mass ions that were significantly expressed on L_3 cuticle or sheath surface.

MCR analysis – negative polarity

Images for highly loaded mass ions for MCR component 1- 4 are shown in Figures S7-10.

Glass Substrate –Analysis of the highly loaded mass ions on MCR component 3 (glass substrate) indicate the presence of elemental ions, such as O^- (16.00 u) and Cl^- (34.97 u), as well as low mass carbon functional groups including CN- (26.00 u), CNO^- (42.00 u) and C_2H^- (25.01). Additionally, phosphate related secondary ions such as PO_2^- (62.97 u) and PO_3^- (78.96 u) and silicate ion $SiHO_3^-$ (79.97 u) are also observed, which can be anticipated from this surface. The elemental ions, phosphates and silicate are located in greater abundance on the glass substrate, whereas the low molecular weight carbon functional groups are distributed throughout the field of view and may be residues of poly-L-lysine coating (Figure S9).

Poly-L-lysine - The highly loaded carbon nitrogen compounds identified as the CNO^- (42.00 u), CN^- (26.00 u) and $C_2H_2N^-$ (40.02 u) secondary ions are distributed throughout the field of view for the MCR component 4 (Figure S10). These are common mass fragments for poly-L-lysine and it has coated the glass substrate as well as the L_3 cuticle and sheath.

Figure S11 shows MCR residuals scores image and corresponding MCR Component loadings and mass assignments.

MCR analysis – positive polarity

Preliminary screening of the hyperspectral data set with PCA indicated five components that account for distinct variability within the dataset. Based on these findings MCR analysis was conducted using five components. Figure S12 shows false colour heat maps and residuals for highly loaded mass ions for the positive polarity and show MCR 1 & 4 are specific for emerging L_3 cuticle and sheath respectively, whereas MCR components 2, 3 and 5 demonstrate non-specificity to observed surfaces.

Analysis of the loadings plots for the highly loaded mass ions for MCR component 1 Figure S13A reveals the presence of a high degree of $C_xH_xO_x$ (Table S2) chemistry on the surface of the cuticle, of which CH_5O^+ (33.04 u), $C_2H_3O_2^+$ (59.02 u), $C_2H_4O_2^+$, $C_3H_7O_2$+ (75.05 u), demonstrate specificity, Figure S14. Although MCR component 4 demonstrate specificity for the sheath, when further analysis is conducted on the highly loaded ions (Figure S13B) none highly loaded compounds are specific for the sheath, Figure S15. This observation was confirmed using the statistical scoring which showed no positive ions are unique of significantly found on the surface of the sheath.

Further statistical analysis

Correlation coefficient analysis and statistical scoring were used to confirm the findings from MCR analysis and to show L_3 cuticle and sheath are chemical different entities. It is important to note these methods utilized data from 12 different partially exsheathed *N. americanus*, for which 24 different regions of interest were defined (12 L_3 cuticles and 12 sheaths), whereas MCR analysis was conducted on a hyperspectral dataset for entire fields of view containing a mixture of partially exsheathed helminths, glass substrate and poly-L-lysine.

Correlation coefficient analysis - Calculation of the correlation coefficients between two variables, the *N. americanus* L_3 cuticle and sheath, permits interpretation of their linear interdependence, based on the coefficients sign (±) and strength (-1< correlation coefficient >1). As expected, all coefficients calculated for this data set are positive. This is because the data collected describes the presence and count for mass ions from a peak list rather than an absolute decrease from predetermined quantities. However, analysis of the strength of the correlation between *N. americanus* L_3 cuticle and sheath surfaces indicates there are differences between their corresponding normalised m/z ions counts, Figure S16. This is implied in regions comparing the L_3 cuticle and sheath, where the average correlation coefficient is 0.85 ± 0.09, whereas regions comparing L_3 cuticle and L_3 cuticle or sheath and sheath are 0.90 ±0.08 and 0.87 ± 0.12, respectively. Further analysis of the data-set suggest the correlation coefficients for the positive data set comparing L_3 cuticle and sheath positive ions are weak and could be the source of chemical differences,. It is important to note when there are subtle differences in the correlation coefficients, as described in this example, it is challenging to isolate sources of variance. Bearing this in mind MCR (main article) and PCA analysis (below) was applied to data set to isolate components that have the strongest anti-correlation or variance.

Statistical Scoring - To refine the data set further, masses that were significantly expressed on either L_3 cuticle or sheath surfaces were identified by combining data filters with Student's t-test. Data filters were applied to highlight masses with a minimum of twofold greater abundance on either the L_3 cuticle or sheath, whilst Student's t-test was performed to confirm the statistical significance ($p<0.01$) Based on the data processing described above 60 (7.3% of all masses surveyed) and 21 (2.6% of all masses surveyed) mass ions were recorded to be unique or were significantly expressed on the L_3 cuticle and sheath, respectively (Table S3).

Normalised mass intensities shows representative mass spectra for mass ions for the L_3 cuticle (152.98 u, 33.04 u, 75.05 u Figure S17 A-C) and corresponding sheath (166.9 u, 178.84 u and 225.01 u Figure S17 D-F) that have demonstrated comparatively significant greater abundance, after analysing PC Loadings, data filters and statistical tests. For example, 152.99 u and 166.97 u have p values of 2.06×10^{-6} and 1.54×10^{-6} (n=12), respectively.

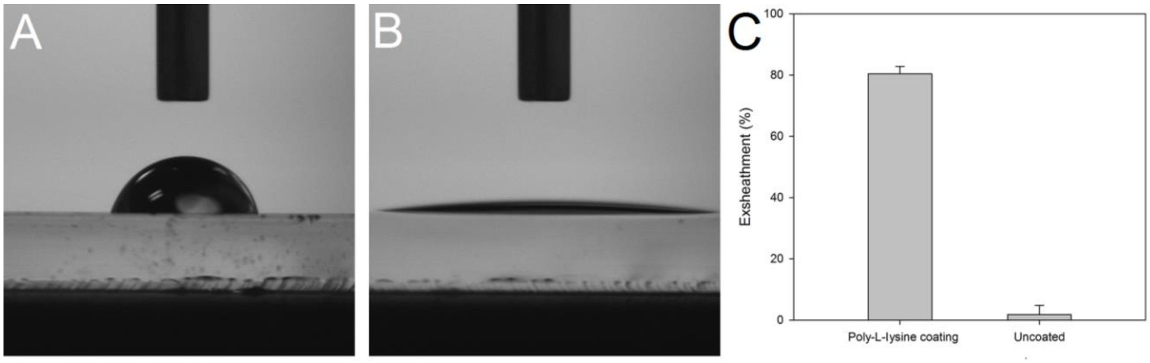

Figure S1. Water contact angle analysis for (A) poly-L-lysine coated, 67.27 ± 5.33°, and (B) uncoated slides, 5.15 ± 1.09° (n=3). (C) Percentage of exsheathed larva deposited on either poly-L-lysine coated glass slides (n=3, larva=20)

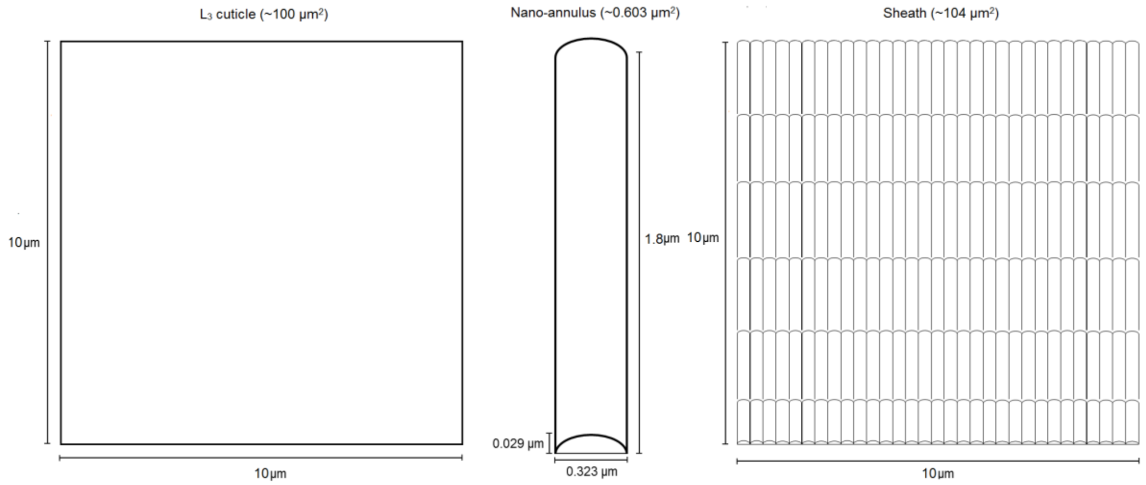

Figure S2. Illustration of surface structure of L_3 cuticle, nano-annulus and sheath with approximate surface area calculations

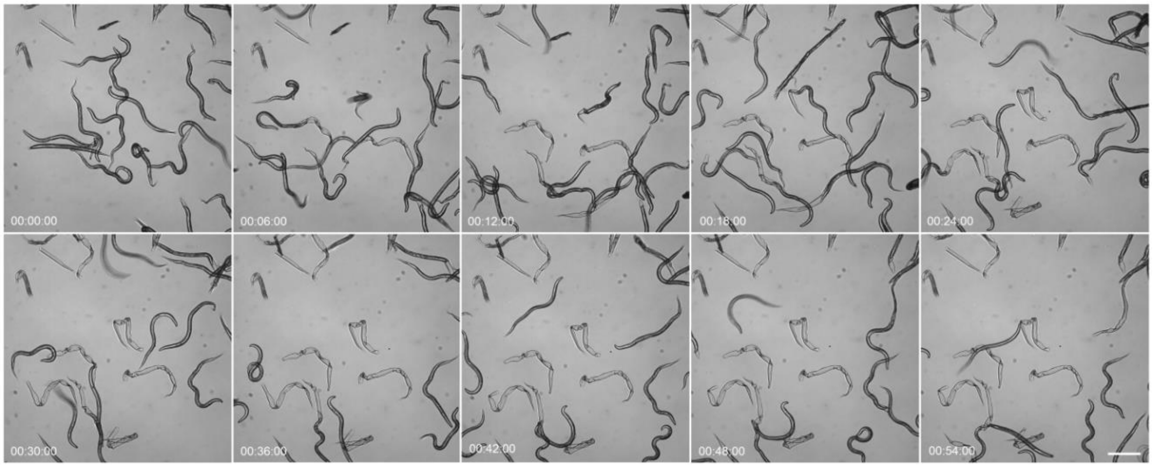

Figure S3. Time-lapse images of L_3 N. americanus exsheathing on poly-L-lysine coated glass slides (37 °C). Scale bar = 200 μm.

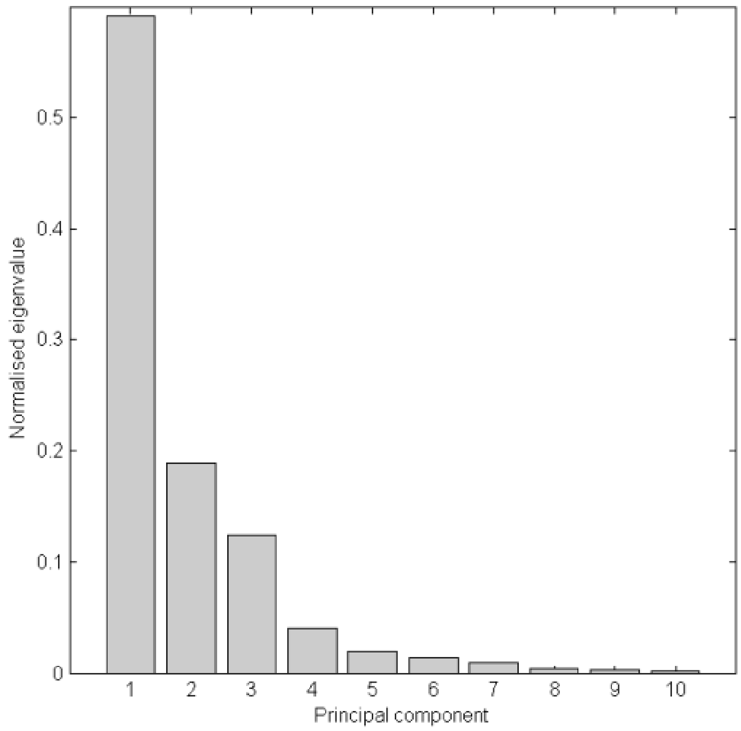

Figure S4. Normalised eigenvalues for L_3 cuticle (n=12) and sheath (n=12).

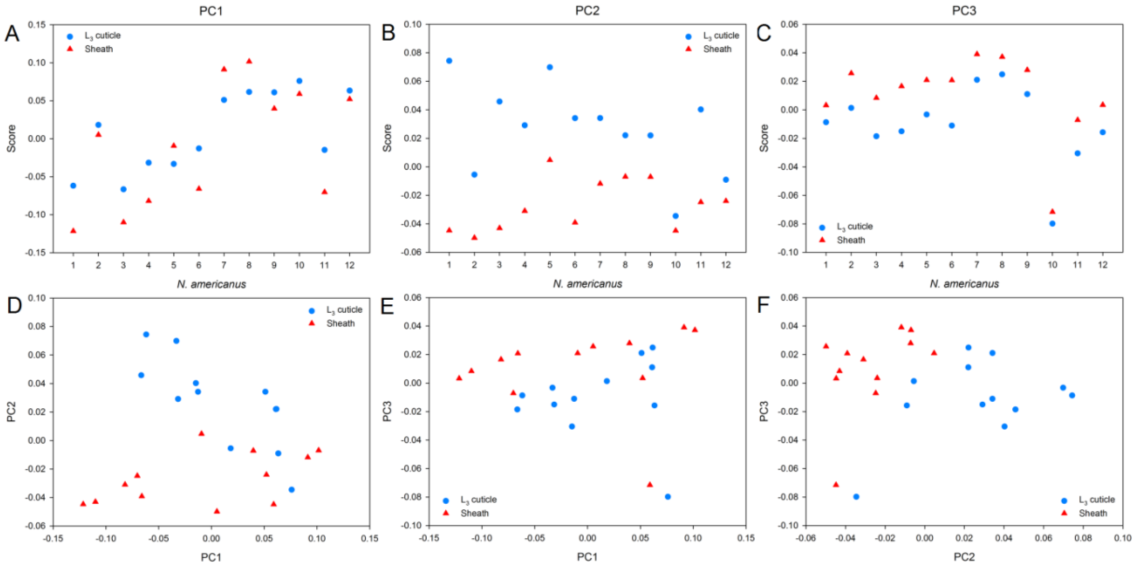

Figure S5. Mean centred scores plot for (A) PC1, (B) PC2 and (C) PC3 alone. Comparison of scores for (D) PC1 & PC2, (D) PC1 & PC3 and (F) PC2 & PC3.

.

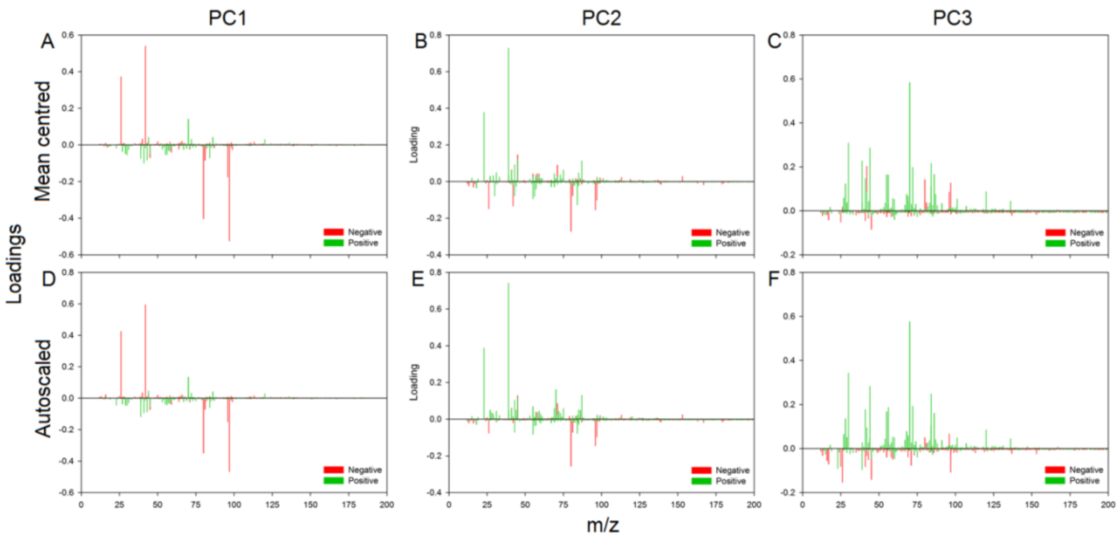

Figure S6. Comparison of coefficients of principal component 1, 2 and 3 for positive (green) and negative (red) mass spectra. (A-C) Mean centred and (D-F) auto-scaled loadings.

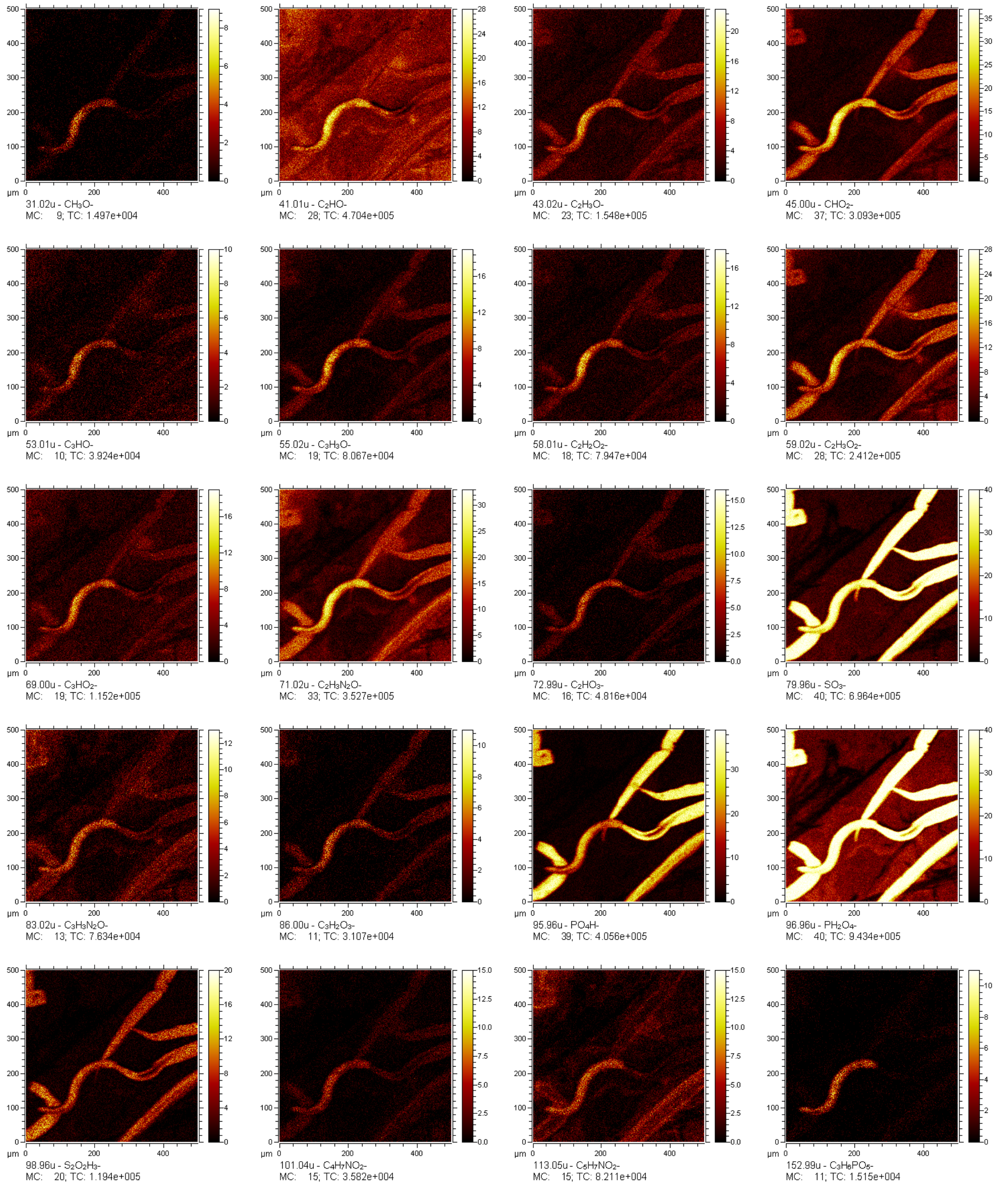

Figure S7. Images for highly loaded mass ions for MCR component 1 (cuticle).

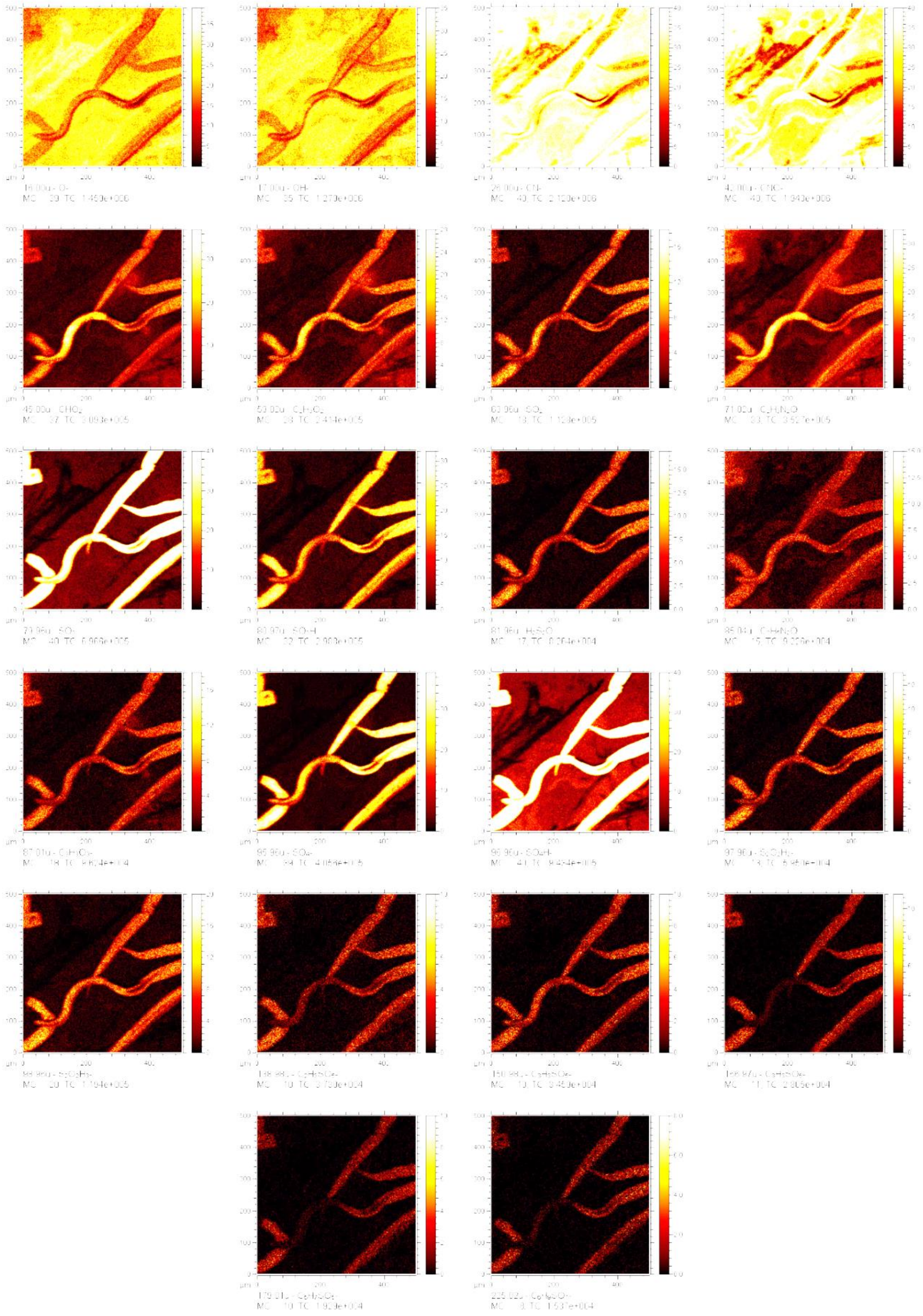

Figure S8. Images for highly loaded mass ions for MCR component 3 (sheath).

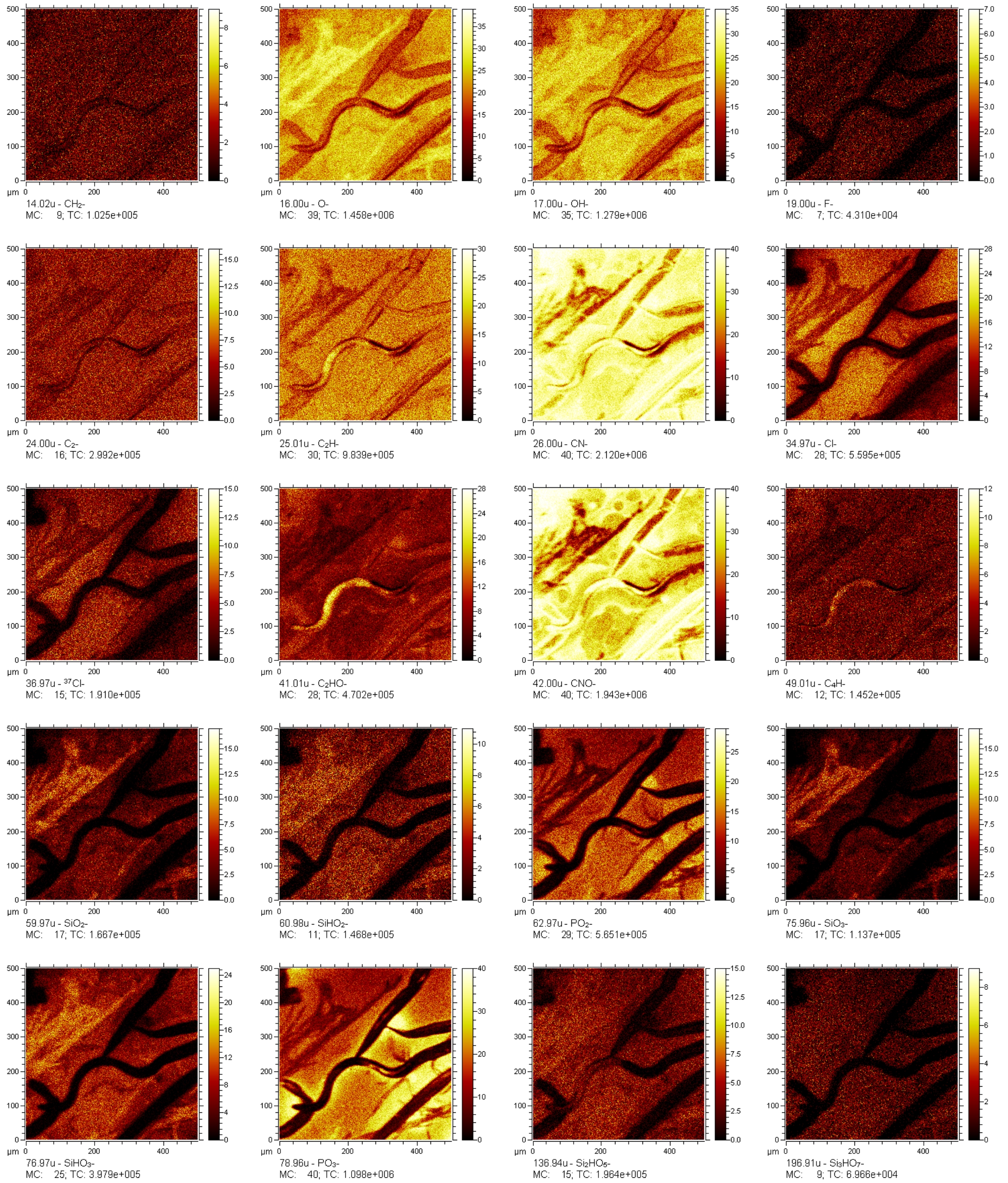

Figure S9. Images for highly loaded mass ions for MCR component 2 (glass substrate).

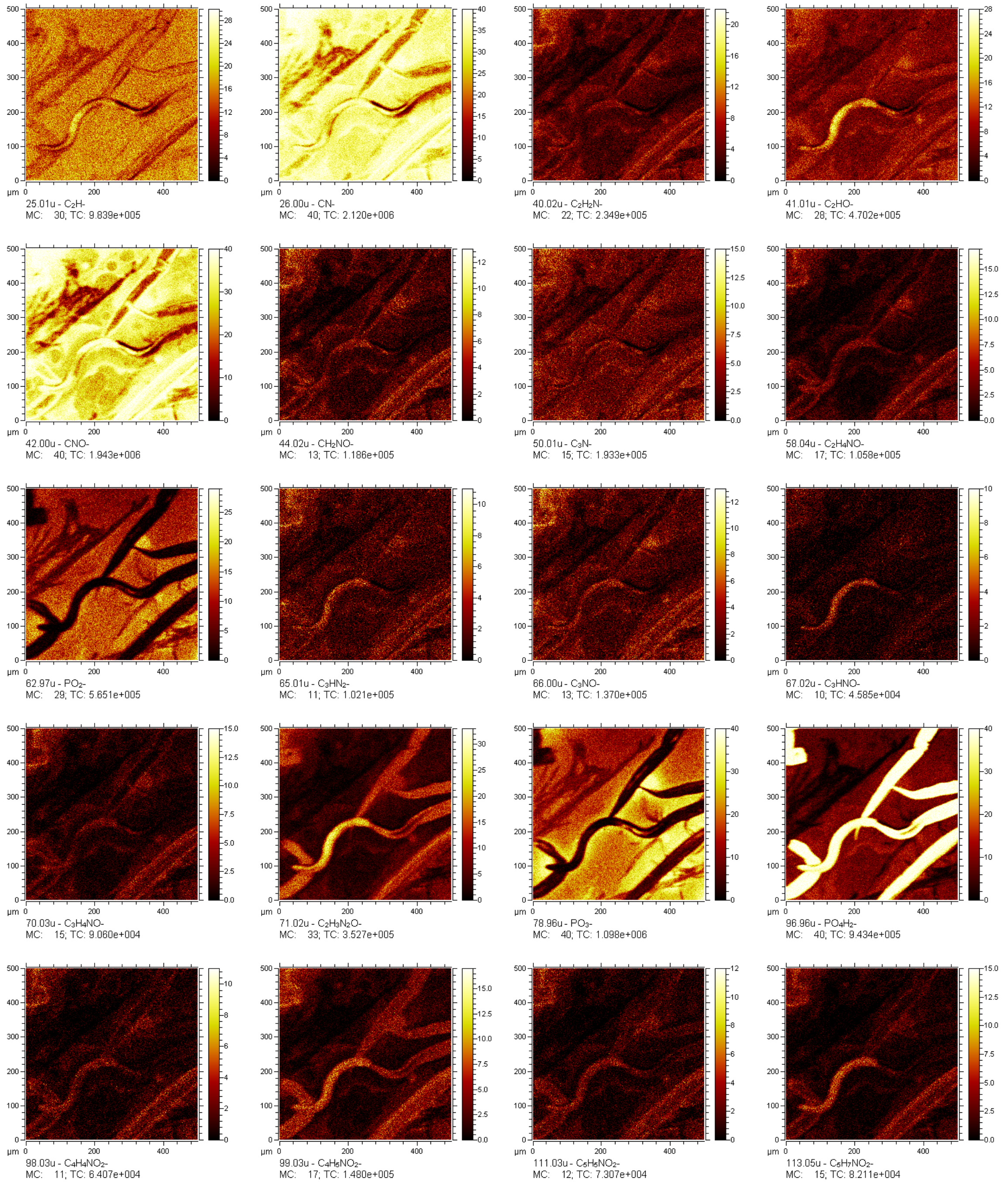

Figure S10. Images for highly loaded mass ions for MCR component 4 (poly-L-lysine).

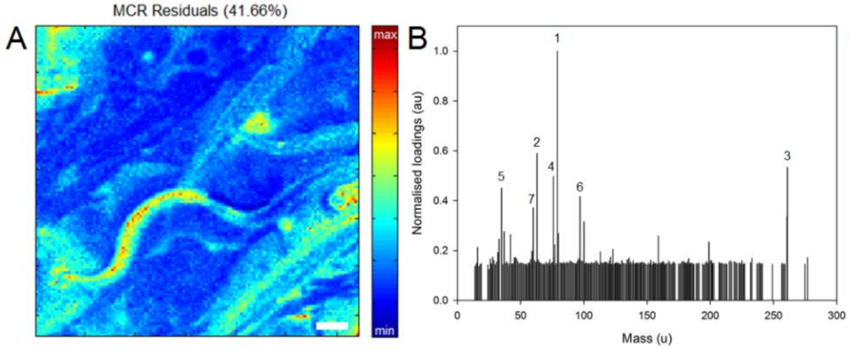

C

	Mass (u)	Assignment
1	78.96	PO_3-
2	62.97	PO_2-
3	260.87	$Na_3S_2O_8$-
4	75.96	SiO_3-
5	34.97	Cl-
6	96.96	SO_4H-
7	59.97	SiO_2-

Figure S11. MCR residuals (A) scores image, (B) MCR Component loadings and (C) mass assignments.

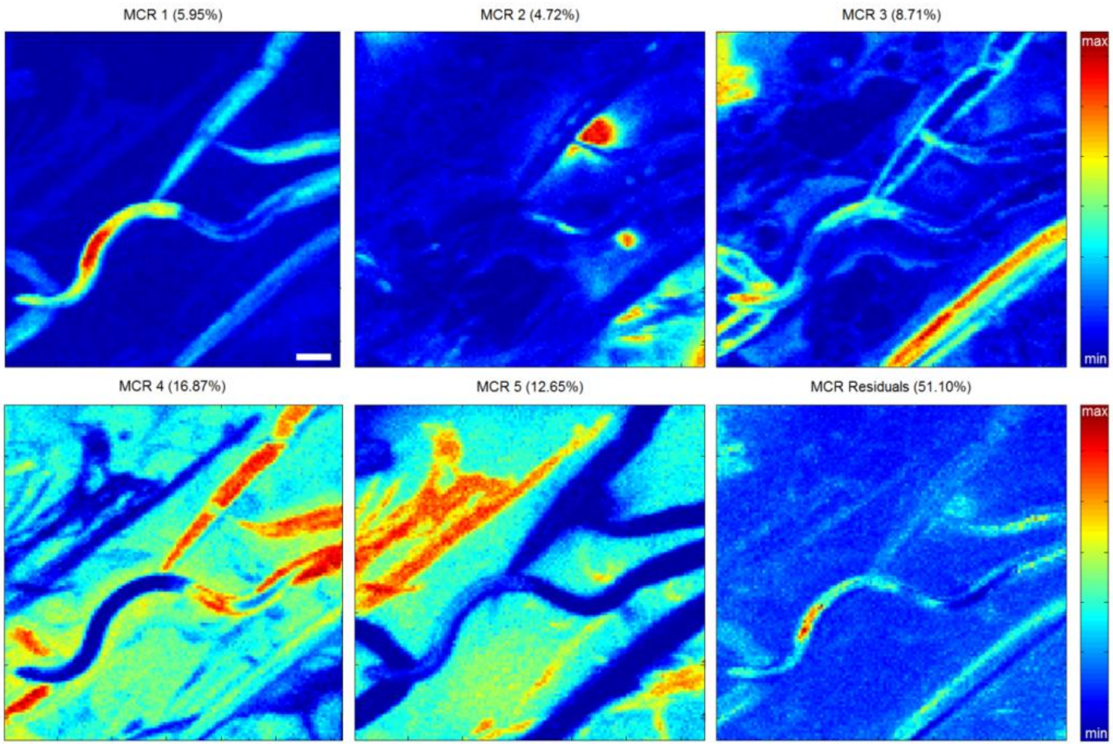

Figure S12. False colour heat maps for highly loaded mass ions for positive polarity MCR components 1-5 and residuals. MCR 1 & 4 demonstrate specify for the emerging hookworm cuticle and sheath, respectively, whereas MCR 2, 3 & 5, demonstrate non-specificity to surfaces.

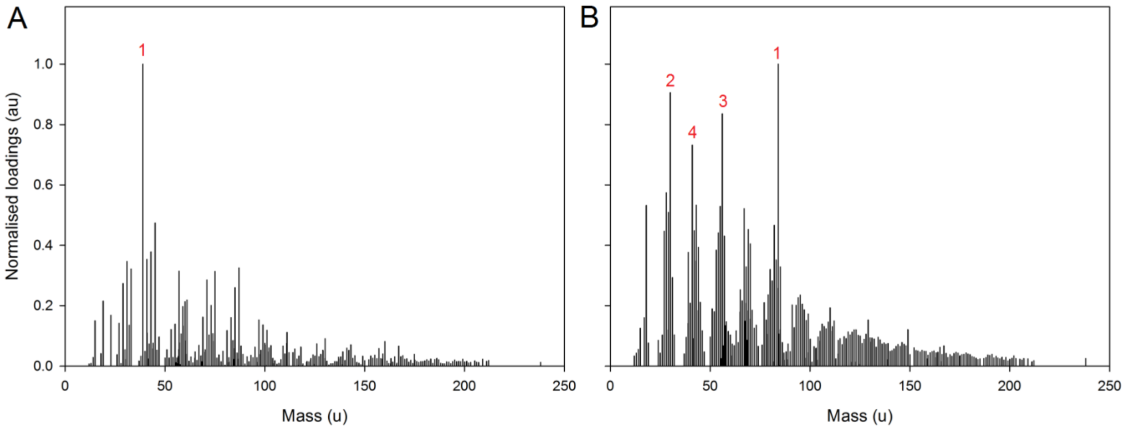

Figure S13. Positive polarity loadings for MCR (A) component 1 (cuticle) and (B) component 4 sheath

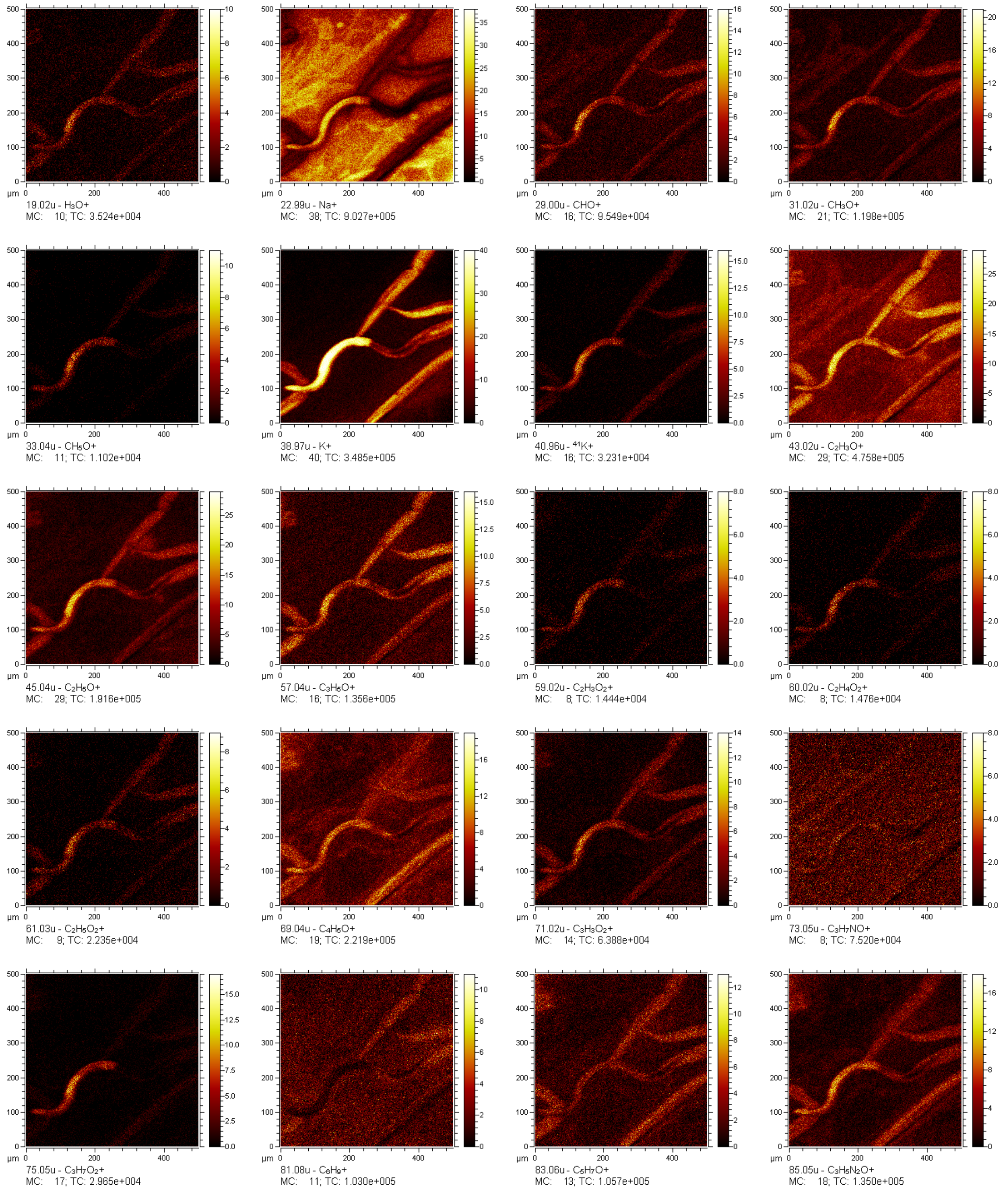

Figure S14. Images for highly loaded mass ions for positive polarity ToF-SIMS MCR component 1 (cuticle).

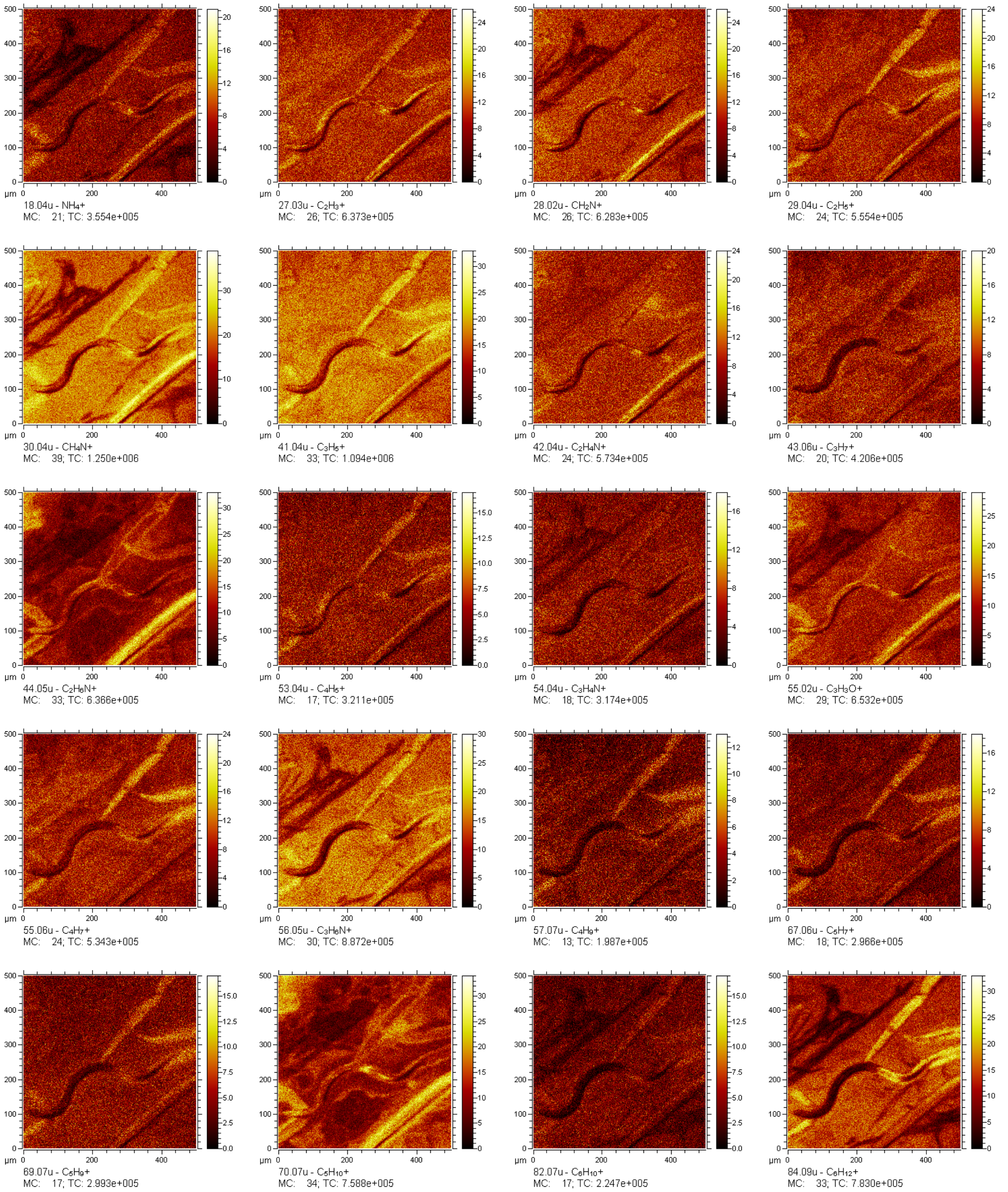

Figure S15. Images for highly loaded mass ions for positive polarity ToF-SIMS MCR component 4 (sheath).

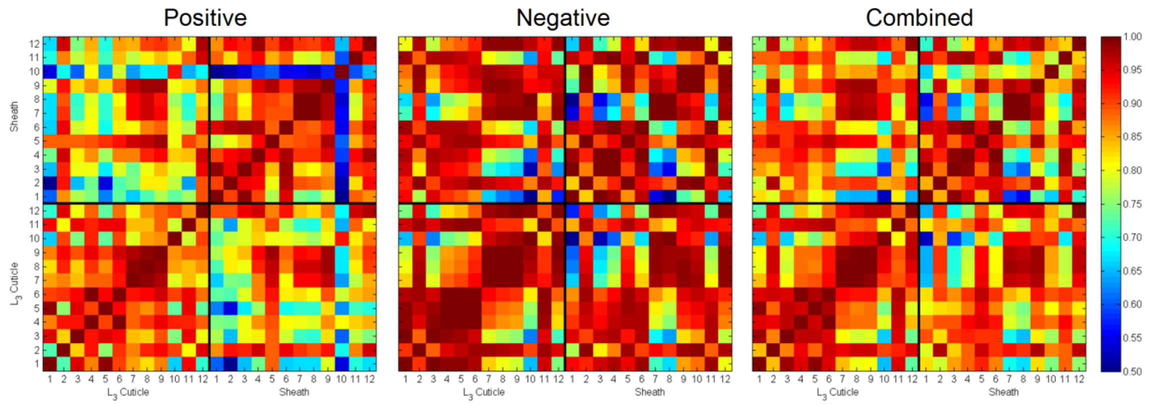

Figure S16. Correlation covariance matrices for positive, negative and combined mass spectra for L_3 cuticle and sheath regions of interest.

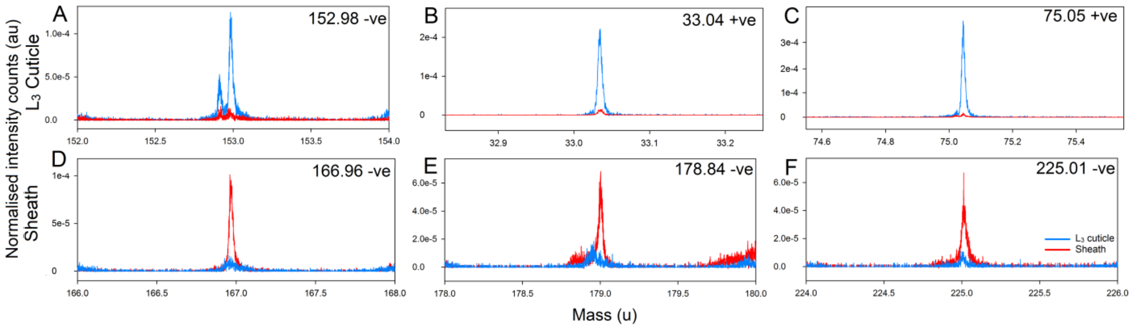

Figure S17. Representative normalised mass spectra for mass ions that show significant abundance on the L_3 cuticle (A-C) and sheath (D-F).Mass spectra are normalised to total mass count and $p<0.01$.

Table S1. Top loaded masses for MCR components 1-4, sorted according to Mass (u)

MCR Component 1			
Rank	Mass (u)	Assignments	Deviation (ppm)
12	31.02	CH_3O-	5.82
13	41.01	C_2HO-	66.17
8	43.02	C_2H_3O-	68.17
1	45.00	CHO_2-	76.25
18	53.01	C_3HO-	58.62
4	55.02	C_3H_3O-	47.56
5	58.01	$C_2H_2O_2$-	36.48
6	59.02	$C_2H_3O_2$-	79.34
10	69.00	C_3HO_2-	18.34
3	71.02	$C_2H_3N_2O$-	-46.10
11	72.99	C_2HO_3-	9.82
7	79.96	SO_3-	31.96
19	83.02	$C_3H_3N_2O$	-27.70
16	86.00	$C_3H_2O_3$-	16.20
15	95.96	PO_4H-	-62.70
9	96.96	PH_2O_4-	-61.71
20	98.96	$S_2O_2H_3$-	10.17
14	101.04	$C_4H_7NO_2$-	-84.43
17	113.05	$C_5H_7NO_2$-	2.26
2	152.99	$C_3H_6PO_5$-	-67.21

MCR Component 2			
Rank	Mass (u)	Assignment	Deviation (ppm)
21	16.00	O-	59.42
18	17.00	OH-	44.20
14	26.00	CN-	46.30
16	42.00	CNO-	98.01
6	45.00	CHO_2-	76.25
8	59.02	$C_2H_3O_2$-	78.98
10	63.96	SO_2-	16.77
11	71.02	$C_2H_3N_2O$-	-46.10
2	79.96	SO_3-	31.93
4	80.97	SO_3H-	18.63
7	81.96	H_2S_2O-	6.89
19	85.04	$C_3H_5N_2O$-	-15.10
9	87.01	$C_3H_3O_3$-	43.69
1	95.96	SO_4-	36.46
3	96.96	SO_4H-	36.43
12	97.96	$S_2O_2H_2$-	59.39
5	98.96	$S_2O_2H_3$-	10.17
15	138.98	$C_2H_3SO_5$-	50.76
17	150.98	$C_3H_3SO_5$-	41.89
13	166.97	$C_3H_3SO_6$-	41.92
20	179.01	$C_5H_7SO_5$-	28.60
22	225.02	$C_6H_9SO_7$-	64.57

MCR Component 3			
Rank	Mass (u)	Assignment	Deviation (ppm)
18	14.02	CH_2-	16.45
1	16.00	O-	59.42
2	17.00	OH-	44.16
19	19.00	F-	2.73
15	24.00	C_2-	-20.68
8	25.01	C_2H-	-5.07
3	26.00	CN-	46.30
4	34.97	Cl-	6.35
12	36.97	^{37}Cl-	7.24
16	41.01	C_2HO-	66.11
7	42.00	CNO-	98.01
20	49.01	C_4H-	30.53
10	59.97	SiO_2-	13.19
13	60.98	$SiHO_2$-	10.61
9	62.97	PO_2-	49.89
14	75.96	SiO_3-	-13.38
5	76.97	$SiHO_3$-	6.54
6	78.96	PO_3-	50.97
11	136.94	Si_2HO_5-	-13.20
17	196.91	Si_3HO_7-	10.39

MCR Component 4			
Rank	Mass (u)	Assignment	Deviation (ppm)
12	25.01	C_2H-	-5.07
2	26.00	CN-	46.30
4	40.02	C_2H_2N-	48.54
8	41.01	C_2HO-	66.11
1	42.00	CNO-	98.01
7	44.02	CH_2NO-	74.55
13	50.01	C_3N-	41.40
5	58.04	C_2H_4NO-	89.95
11	62.97	PO_2-	49.89
19	65.01	C_3HN_2-	-40.20
10	66.00	C_3NO-	29.73
20	67.02	C_3HNO-	130.39
6	70.03	C_3H_4NO-	69.12
14	71.02	$C_2H_3N_2O$-	-46.06
3	78.96	PO_3-	50.97
9	96.96	PO_4H_2-	-61.71
17	98.03	$C_4H_4NO_2$-	28.68
15	99.03	$C_4H_5NO_2$-	-46.42
18	111.03	$C_5H_5NO_2$-	3.73
16	113.05	$C_5H_7NO_2$-	2.26

Table S2. Top loaded masses for positive polarity ToF-SIMS MCR components 1 and 4, sorted according to Mass (u)

MCR Component 1 (Cuticle)			
Rank	Assignment	Mass (u)	Deviation
14	H_3O^+	19.02	55.26
18	Na^+	22.99	103.17
11	CHO^+	29.00	74.28
5	CH_3O^+	31.02	81.03
7	CH_5O^+	33.04	84.68
1	K^+	38.97	83.43
4	$^{41}K^+$	40.96	54.30
3	$C_2H_3O^+$	43.02	75.17
2	$C_2H_5O^+$	45.04	64.87
8	$C_3H_5O^+$	57.04	65.07
17	$C_2H_3O_2^+$	59.02	58.54
15	$C_2H_4O_2^+$	60.02	46.50
13	$C_2H_5O_2^+$	61.03	79.45
19	$C_4H_5O^+$	69.04	89.20
10	$C_3H_3O_2^+$	71.02	72.02
16	$C_3H_7NO^+$	73.05	31.83
9	$C_3H_7O2^+$	75.05	128.92
20	$C_6H_9^+$	81.08	105.94
12	$C_5H_7O^+$	83.06	85.73
6	$C_3H_5N_2O^+$	85.05	106.95

MCR Component 4 (Sheath)			
Rank	Assignment	Mass (u)	Deviation
7	NH_4^+	18.04	78.27
15	$C_2H_3^+$	27.03	91.48
5	CH_2N^+	28.02	113.19
11	$C_2H_5^+$	29.04	99.96
2	CH_4N^+	30.04	123.56
4	$C_3H_5^+$	41.04	70.44
14	$C_2H_4N^+$	42.04	107.74
6	$C_3H_7^+$	43.06	66.49
19	$C_2H_6N^+$	44.05	89.11
20	$C_4H_5^+$	53.04	48.33
16	$C_3H_4N^+$	54.04	93.61
10	$C_3H_3O^+$	55.02	78.29
8	$C_4H_7^+$	55.06	63.57
3	$C_3H_6N^+$	56.05	93.11
17	$C_4H_9^+$	57.07	81.33
9	$C_5H_7^+$	67.06	57.63
13	$C_5H_9^+$	69.07	65.91
18	$C_5H_{10}^+$	70.07	-50.21
12	$C_6H_{10}^+$	82.07	-36.23
1	$C_6H_{12}^+$	84.09	12.16

Table S3. Mass ions identified and ranked as having significant abundance on either L_3 cuticle or sheath ($p<0.01$, $n=12$). Masses highlighted in red and green refer to negative and positive mass spectra, respectively.

L_3 Cuticle						Sheath		
Rank	Mass unit (u)	P-value	Rank	Mass unit (u)	P-value	Rank	Mass unit (u)	P-value
1	154.98	3.69E-08	31	23.99	1.75E-04	1	180.03	1.24E-10
2	134.98	2.42E-07	32	58.96	1.97E-04	2	389.09	1.48E-08
3	257.03	5.06E-07	33	24.99	2.14E-04	3	535.16	8.03E-08
4	255.02	6.35E-07	34	254.85	2.91E-04	4	243.01	5.17E-07
5	97.11	7.44E-07	35	75.06	3.57E-04	5	166.96	1.54E-06
6	239.02	1.21E-06	36	158.92	3.81E-04	6	663.17	2.99E-06
7	273.03	1.84E-06	37	33.04	4.00E-04	7	647.34	3.15E-06
8	79.02	2.02E-06	38	75.05	4.60E-04	8	138.98	5.05E-06
9	152.98	2.06E-06	39	447.06	6.04E-04	9	178.96	5.83E-06
10	136.99	2.11E-06	40	76.07	6.86E-04	10	178.84	5.91E-06
11	88.02	2.41E-06	41	84.97	6.99E-04	11	162.95	9.50E-06
12	267.02	3.33E-06	42	59.01	8.09E-04	12	533.10	1.16E-05
13	137.82	5.45E-06	43	56.97	1.05E-03	13	179.93	1.27E-05
14	271.01	5.87E-06	44	60.02	1.08E-03	14	517.13	1.42E-05
15	69.00	9.80E-06	45	118.94	1.13E-03	15	225.01	1.71E-05
16	101.03	1.14E-05	46	74.02	1.31E-03	16	395.42	4.25E-05
17	315.03	1.97E-05	47	110.98	1.36E-03	17	501.13	9.17E-05
18	31.02	2.00E-05	48	39.96	1.71E-03	18	387.08	1.92E-04
19	55.02	2.04E-05	49	142.95	2.26E-03	19	545.10	4.58E-04
20	136.93	2.51E-05	50	270.83	2.28E-03	20	515.07	1.33E-03
21	345.05	2.55E-05	51	73.03	2.56E-03	21	531.15	1.39E-03
22	100.01	2.58E-05	52	45.03	2.69E-03			
23	53.00	2.84E-05	53	32.02	3.12E-03			
24	154.90	4.13E-05	54	101.03	3.14E-03			
25	40.96	4.70E-05	55	61.04	3.22E-03			
26	58.01	4.94E-05	56	87.96	3.28E-03			
27	103.05	6.91E-05	57	401.08	3.56E-03			
28	22.99	9.83E-05	58	170.88	5.10E-03			
29	238.87	1.09E-04	59	58.99	6.43E-03			
30	70.98	1.56E-04	60	19.02	7.23E-03			

Other Supporting Information for this manuscript includes the following:

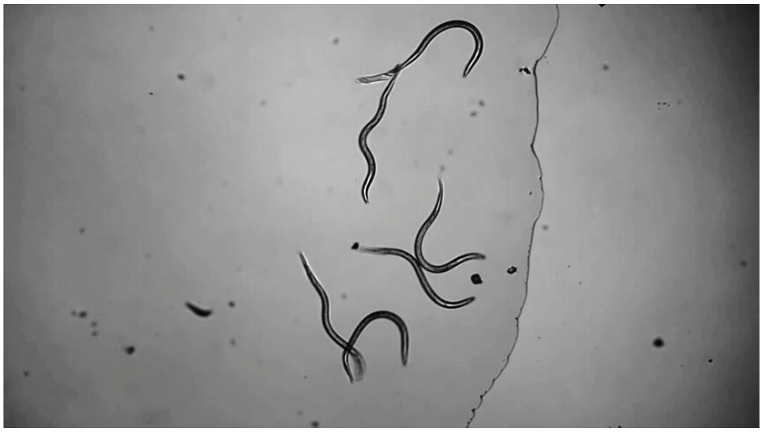

S1 Movie. Exsheathment behaviour of *N. americanus* at 37 °C in the absence of poly-L-lysine coated glass substrate.

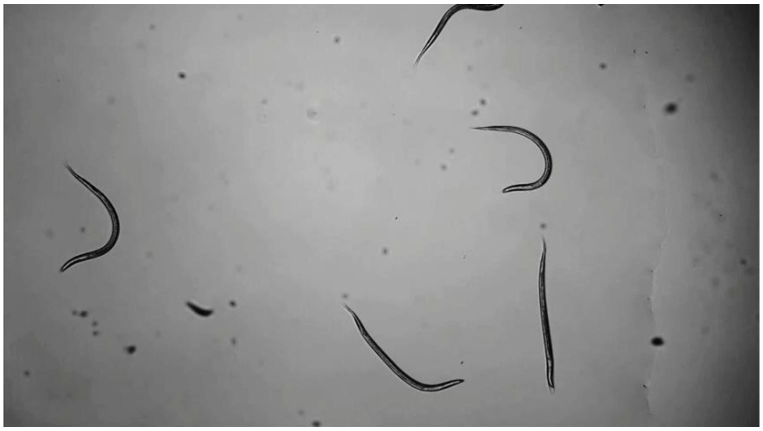

S2 Movie. Exsheathment behaviour of *N. americanus* at 20 °C in the absence of poly-L-lysine coated glass substrate.

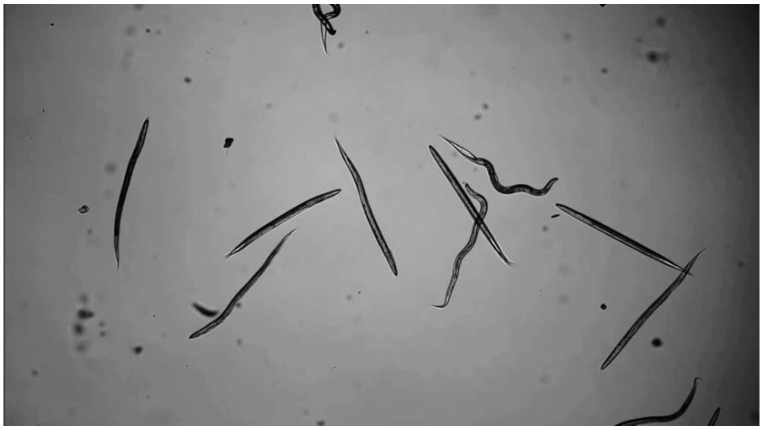

S3 Movie. Exsheathment behaviour of *N. americanus* at 20 °C in the presence of poly-L-lysine coated glass substrate. This video was captured using an inverted optical microscope and shows a mobile larva on top of stationary larva. The mobile larva is able to move the centre of its anatomy, because it is only in partial in contact with the poly-L-lysine surfaces at the head and tail. Whereas full the length of stationary larva is in contact with the coated surface and as a result its movement is restricted.

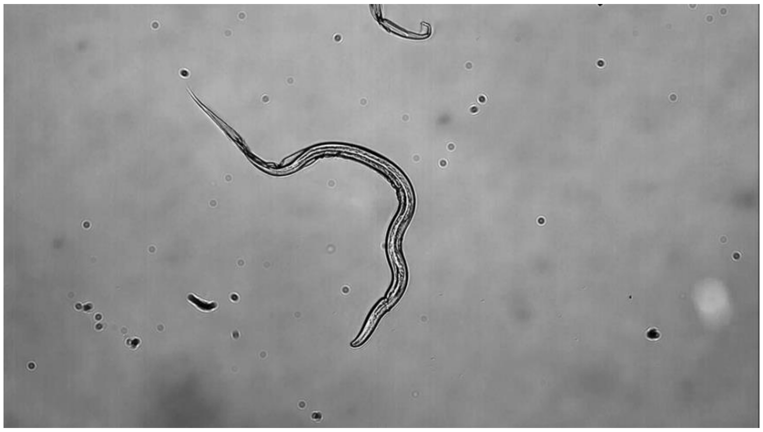

S4 Movie. Exsheathment behaviour of *N. americanus* at 37 °C in the presence of poly-L-lysine coated glass substrate (single larval exsheathment).

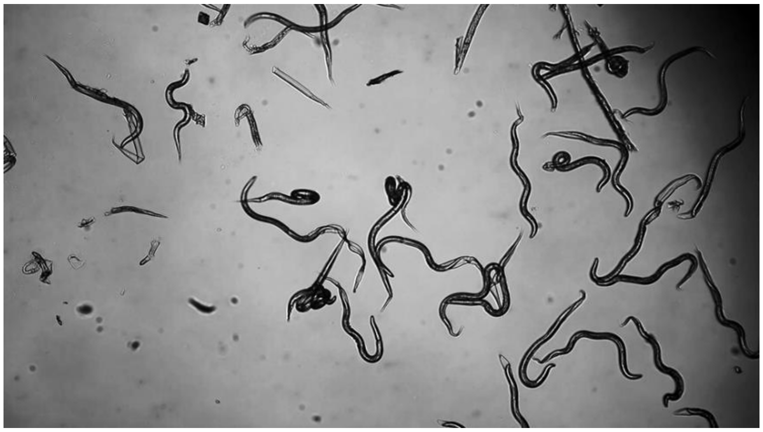

S5 Movie. Exsheathment behaviours of *N. americanus* at 37 °C in the presence of poly-L-lysine coated glass substrate (mass exsheathment).
